# Hif-1α regulates macrophage-endothelial interactions during blood vessel development in zebrafish

**DOI:** 10.1038/ncomms15492

**Published:** 2017-05-19

**Authors:** Claudia Gerri, Rubén Marín-Juez, Michele Marass, Alora Marks, Hans-Martin Maischein, Didier Y R. Stainier

**Affiliations:** 1 Department of Developmental Genetics, Max Planck Institute for Heart and Lung Research, 61231 Bad Nauheim, Germany

## Abstract

Macrophages are known to interact with endothelial cells during developmental and pathological angiogenesis but the molecular mechanisms modulating these interactions remain unclear. Here, we show a role for the Hif-1α transcription factor in this cellular communication. We generated *hif-1aa*;*hif-1ab* double mutants in zebrafish, hereafter referred to as *hif-1α* mutants, and find that they exhibit impaired macrophage mobilization from the aorta-gonad-mesonephros (AGM) region as well as angiogenic defects and defective vascular repair. Importantly, macrophage ablation is sufficient to recapitulate the vascular phenotypes observed in *hif-1α* mutants, revealing for the first time a macrophage-dependent angiogenic process during development. Further substantiating our observations of vascular repair, we find that most macrophages closely associated with ruptured blood vessels are Tnfα-positive, a key feature of classically activated macrophages. Altogether, our data provide genetic evidence that Hif-1α regulates interactions between macrophages and endothelial cells starting with the mobilization of macrophages from the AGM.

## Abstract (web summary)

The molecular mechanism regulating macrophage interaction with endothelial cells during development is unclear. Here, the authors show that in zebrafish mutation of hypoxia-inducible factor-1α impairs macrophage mobilization from the aorta-gonad-mesonephros, causing defects in angiogenesis and vessel repair.

Macrophages are widely known as important elements of innate immunity, taking part in the first line of defence against pathogen invasion. Besides their role in immunity, macrophages are involved in several processes that occur independently of immune cell signalling. They can secrete growth factors and cytokines, as well as proteolytic enzymes to remodel the extracellular matrix (ECM), and in doing so play crucial roles in tissue and organ growth and homeostasis^1^.

Previous studies have reported the role of macrophages in supporting angiogenesis. Early work in the guinea pig cornea showed that activated macrophages *in vitro* and *in vivo* induce vascular proliferation^2^. Moreover, the pro-angiogenic functions of macrophages have been studied in several pathological conditions. In these scenarios, macrophages can secrete key pro-angiogenic mediators^3^, and by secreting membrane-bound and soluble proteases they can promote the remodelling of the ECM, providing survival and guidance cues to endothelial cells (ECs)^4^. Particularly, Tnfα, a major secretory product of activated macrophages, has been implicated in different stages of inflammation and wound repair^5^. However, in other pathological settings, such as during tumour growth, Tnfα is considered a double edged sword as it can be both pro- and anti-tumorigenic^6^,^7^,^8^. Recently, some studies have proposed macrophages as modulators of vessel bed formation and fusion of endothelial tip cells during developmental angiogenesis^9^,^10^,^11^. Despite these reports, the molecular mechanisms modulating macrophage-endothelial interactions remain largely unknown.

In addition to macrophages, molecular oxygen (O_2_) is also known to be a potent regulator of cell behaviour^12^; and hypoxia-inducible factors (HIFs) are key effectors of low O_2_ sensing during cellular adaptation^13^,^14^. HIF consists of a heterodimer of an O_2_-regulated α-subunit and a constitutively expressed β-subunit. Under normoxia, HIFα undergoes transcriptional as well as post-translational regulation, the latter modulated by the factor inhibiting HIF (FIH)^15^ and the prolyl hydroxylases (PHDs)/Von-Hippel Lindau tumour suppressor protein (VHL) axis^16^. In hypoxic conditions, PHDs and FIH are inactivated allowing HIFα translocation into the nucleus where, on dimerization with HIF-1β, it regulates the expression of numerous downstream target genes^17^.

Genetic studies in mouse have shown that HIF is essential for vascular development, as deletion of *Hif-1α* or *Hif-1β* impairs the formation of the vascular system causing early embryonic lethality^18^,^19^,^20^,^21^. Moreover, myeloid-specific deletion of *Hif-1α* causes abnormalities in the motility, adhesion and invasiveness of peritoneal macrophages^22^. Interestingly, a recent study has suggested that HIF-1α plays a critical role in macrophages during vascular remodelling after femoral artery injury^23^. However, how HIF-1α regulates macrophage function during vascular development and blood vessel repair remains poorly understood.

In this work, we leverage the zebrafish model to study Hif-1α signalling in a global knockout; zebrafish embryos are hypoxia-tolerant in early stages of development^24^, thereby overcoming the embryonic lethality observed in *Hif-1α* mutant mice. To study the role of *hif-1α* in macrophage-endothelial communication, we generated *hif-1α (hif-1aa;hif-1ab)* zebrafish mutants using TALEN and CRISPR/Cas9 technologies^25^,^26^. Interestingly, *hif-1α* mutants exhibit profound defects in dorsal longitudinal anastomotic vessel (DLAV) plexus formation in normoxic conditions, as well as vessel ruptures after hypoxic stress. Importantly, we find impaired macrophage mobilization from the aorta-gonad-mesonephros (AGM) region in *hif-1α* mutants. In addition, we show that macrophages are required for DLAV plexus formation, a process that fails to occur in *hif-1α* mutant embryos. These data reveal for the first time a direct requirement for macrophages during a developmental angiogenic process. In addition, in wild type (WT) siblings, but not in *hif-1α* mutants, macrophages are able to assist the repair of blood vessel ruptures. Importantly, we observe that most macrophages in close association with ruptured vessels are Tnfα-positive. Altogether, these data provide further insights into the role of Hif-1α during blood vessel development and repair.

## Results

### Generation of *hif-1aa* and *hif-1ab* mutants

**Figure 1:**
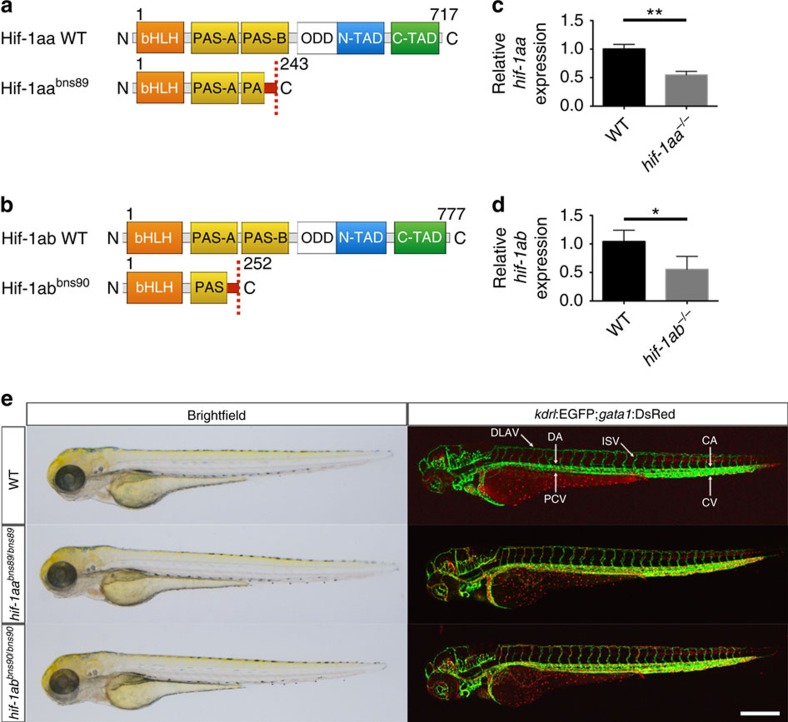
Absence of obvious morphological or vascular defects in *hif-1aa* and *hif-1ab* single mutants. (**a**,**b**) Schematic representation of the following proteins: WT and mutant (bns89 allele) Hif-1aa, WT and mutant (bns90 allele) Hif-1ab. bHLH, basic helix-loop-helix domain; PAS-A/B, PAS domains; ODD, oxygen degradation dependent domain; N-TAD, N-terminal transactivation domain; C-TAD, C-terminal transactivation domain. Red box before the stop codon represents missense sequence. (**c**) qPCR analysis of *hif-1aa* expression in WT siblings and *hif-1aa*^*bns89*^ mutants at 48 hpf. (**d**) qPCR analysis of *hif-1ab* expression in WT siblings and *hif-1ab*^*bns90*^ mutants at 48 hpf. Values represent mean±s.d., *n*=3 biological replicates, (*P<0.05; **P<0.01; *t*-test). (**e**) Representative brightfield images and maximal intensity projections of confocal z-stacks of *Tg(kdrl:EGFP);Tg(gata1:DsRed)* WT siblings and *hif-1aa* and *hif-1ab* mutants at 72 hpf; lateral views. *n*=3 clutches. CA, caudal artery; CV, caudal vein; DA, dorsal aorta; DLAV, dorsal longitudinal anastomotic vessel; ISV, intersegmental vessel; PCV, posterior cardinal vein. Scale bar, 200 μm.

To examine the function of Hif-1α, we generated *hif-1aa* and *hif-1ab* mutant alleles using CRISPR/Cas9 (ref. 26) and TALEN^25^ technologies, respectively. *hif-1aa* mutants were generated targeting a region in exon 6 which encodes the second PAS domain (Fig. 1a). *hif-1ab* mutants were generated targeting a region in exon 6 which encodes the first PAS domain (Fig. 1b). We recovered frame-shift mutant alleles, *hif-1aa*^*bns89*^ and *hif-1ab*^*bns90*^, which have premature stop codons after 13 and 23 amino acid-long missense segments following the lesion site, respectively (Supplementary Fig. 1). To investigate the severity of these mutant alleles, we examined *hif-1aa* and *hif-1ab* transcript levels by quantitative PCR (qPCR) analysis. When compared to WT siblings, *hif-1aa*^*bns89*^ and *hif-1ab*^*bns90*^ mutants display a significant (*t*-test) reduction in the mutated gene's transcript levels of approximately 50% (Fig. 1c,d). To determine whether these mutants exhibit vascular defects, *hif-1aa*^*bns89*^ and *hif-1ab*^*bns90*^ were crossed into the *Tg(kdrl:EGFP)*^*s843*^*;Tg(gata1a:DsRed)*^*sd2*^ lines, hereafter *Tg(kdrl:EGFP);Tg(gata1:DsRed),* which allows visualization of endothelial and erythroid cells, respectively. Confocal imaging confirmed no gross morphological or vascular defects in 72 h post fertilization (hpf) *hif-1aa*^*bns89*^ or *hif-1ab*^*bns90*^mutants (Fig. 1e), which grew to be viable and fertile adults.

### *hif-1α* ^*−/−*^ exhibit angiogenic defects and vascular ruptures

**Figure 2:**
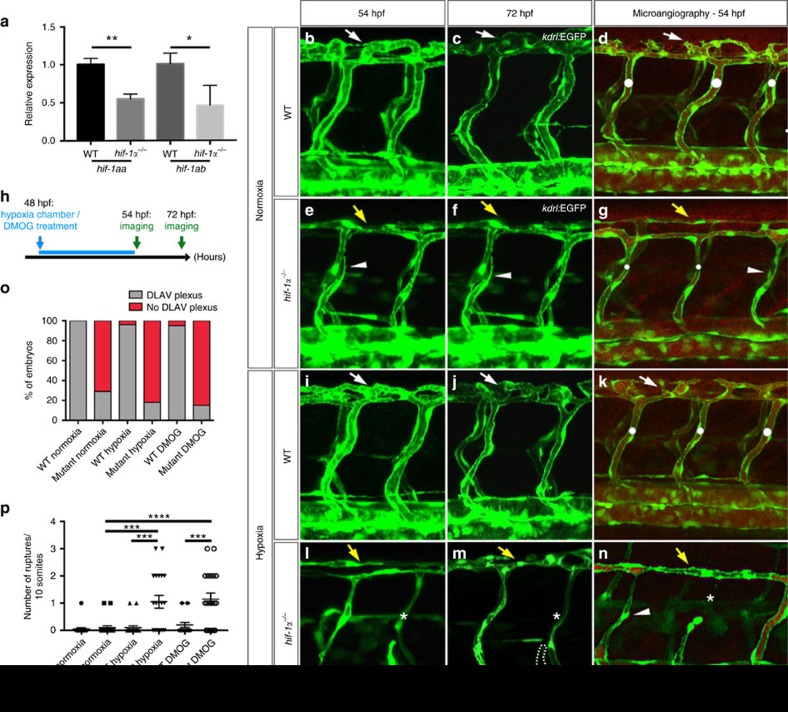
*hif-1α* mutants fail to form the DLAV plexus and exhibit hypoxia-induced vascular disconnections. (**a**) qPCR analysis of *hif-1aa* and *hif-1ab* transcripts in WT siblings and *hif-1α* mutants at 48 hpf. Values represent mean±s.d., *n* = 3 biological replicates, (*P<0.05; **P<0.01; *t*-test). (**b**–**g**) Maximal intensity projections of confocal z-stacks of *Tg(kdrl:EGFP)* WT siblings and *hif-1α*^*−/−*^ embryos in normoxia at 54 and 72 hpf, as well as after microangiography at 54 hpf. (**h**) Schematic representation of the experiment shown in **i**–**p**. (**i**–**n**) Maximal intensity projections of confocal z-stacks of *Tg(kdrl:EGFP)* WT siblings and *hif-1α*^*−/−*^ embryos after hypoxia chamber treatment for 6 h at 54 and 72 hpf, along with microangiography at 54 hpf. All images represent lateral views. White dots indicate approximate lumen size, white arrows point to DLAV plexus in WT, yellow arrows indicate absence of DLAV plexus in *hif-1α*^*−/−*^, arrowheads point to non-perfused ISVs, asterisks indicate vessel ruptures and dotted lines outline regressed blood vessel. (**o**) Quantification of embryos showing normal (grey) or abnormal/absent (red) DLAV plexus formation in 54 hpf WT siblings and *hif-1α*^*−/−*^ in normoxia, and after hypoxia chamber or DMOG treatment for 6 h starting at 48 hpf. (**p**) Quantification of blood vessel ruptures in a 10 somite-long trunk area in 54 hpf WT siblings and *hif-1α*^*−/−*^in normoxia, and after hypoxia chamber or DMOG treatment for 6 h starting at 48 hpf. Bars represent mean±s.e.m., *n*=20 embryos from three different clutches, (***P<0.001; ****P<0.0001; *t*-test). Scale bar, 50 μm.

Suspecting redundancy or compensation^27^, we generated *hif-1aa*^*bns89*^*;hif-1ab*^*bns90*^ double mutants, hereafter abbreviated as *hif-1α* mutants (*hif-1α*^*−/−*^). qPCR analysis showed a significant (*t*-test) reduction of *hif-1aa* and *hif-1ab* transcript levels in *hif-1α*^*−/−*^compared to WT siblings (Fig. 2a). To further validate these mutants, we performed transcriptomic analysis of WT and mutant siblings at 50 hpf in both normoxic and hypoxic conditions. Our qPCR data show that well-known Hif-1α target genes including *phd3*, *pfkfb3* and *hbbe3* were strongly down-regulated in *hif-1α* mutants compared to WT animals (Supplementary Fig. 2a–c), indicating that Hif-1α signalling is efficiently blocked in *hif-1α* mutants. Next, we analysed vascular development in *hif-1α* mutants at 54 hpf. *hif-1α*^*−/−*^ exhibited mild perfusion defects in trunk intersegmental vessels (ISVs) and lack of DLAV plexus formation in normoxic conditions (Fig. 2b,c,e,f). The DLAV plexus develops by angiogenesis between 36 and 96 hpf^28^. Microangiography analysis of *hif-1α*^*−/−*^ confirmed that ISVs had a reduced lumen size and in some cases also a complete lack of perfusion (Fig. 2d,g). Moreover, DLAVs appeared perfused but exhibited a complete absence of plexus formation (Fig. 2d,g). However, when we incubated 24 hpf *hif-1α*^*−/−*^ embryos in hypoxia for 6 h, we did not observe defects in ISV sprouting (Supplementary Fig. 3a,b). Interestingly, previous studies have reported that 48 hpf is a critical stage for zebrafish embryos, representing the transition from a hypoxia-tolerant to a hypoxia-sensitive state^24^. To test this model, we challenged the embryos with hypoxic conditions, using a hypoxia chamber or dimethyloxalylglycine (DMOG), a pan-hydroxylase inhibitor which mimics hypoxia by stabilizing Hifα proteins and thus activating downstream effectors of the pathway^29^,^30^. While WT siblings remained unaffected, *hif-1α*^*−/−*^ embryos challenged by hypoxia chamber or DMOG administration at 48 hpf for 6 h (Fig. 2h), displayed vascular ruptures affecting the connections between the ISVs and dorsal aorta (DA), as well as in the DLAV (Fig. 2i,l and Supplementary Fig. 4). Interestingly, vessels that appeared disconnected at 54 hpf were absent by 72 hpf, probably due to vessel regression (Fig. 2l,m). Microangiography of *hif-1α*^*−/−*^ revealed that under hypoxic stress, vessel perfusion defects were exacerbated, as lack of flow was observed not only in ISVs but also in the DLAV (Fig. 2k,n). Moreover, quantification of these vascular defects confirmed that while both are caused by the lack of *hif-1α* function, DLAV plexus formation is a hypoxia-independent process (Fig. 2o), whereas blood vessel ruptures are observed only after hypoxic treatment (Fig. 2p). In addition, when we incubated 58 hpf *hif-1α*^*−/−*^ embryos in hypoxia for 14 h, the vascular phenotypes did not appear more severe than those observed in 54 hpf *hif-1α*^*−/−*^ embryos (Supplementary Fig. 3c,d). Altogether, these observations reveal that the absence of *hif-1α* function leads to severe defects in DLAV plexus formation as well as hypoxic stress-induced vascular ruptures.

### *hif-1α* is required for macrophage mobilization from the AGM

**Figure 3:**
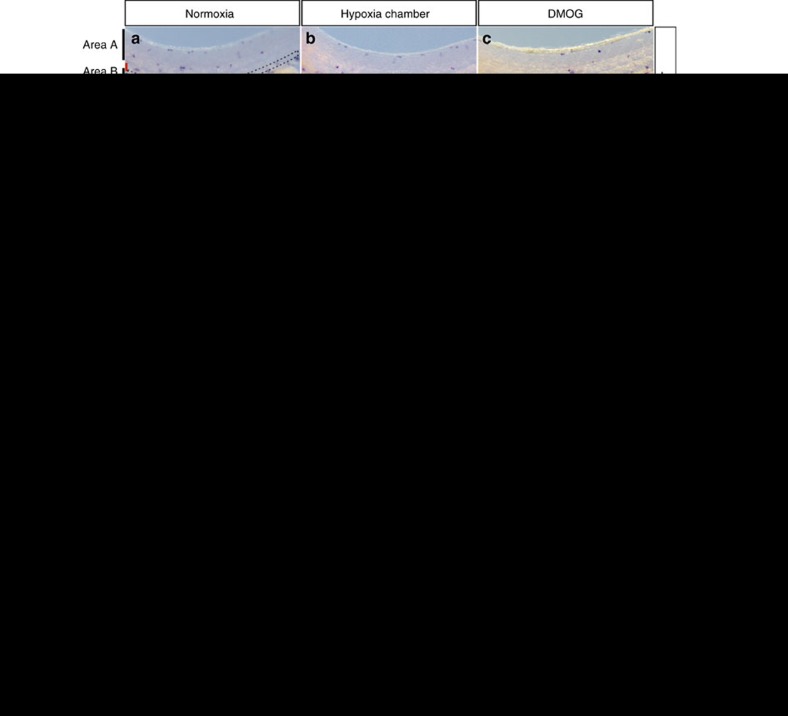
*hif-1α* is required for macrophage mobilization from the AGM region. (**a**–**f**) Brightfield images of WISH for *mfap4* expression in 54 hpf WT siblings and *hif-1α*^*−/−*^ in normoxia, and after hypoxia chamber or DMOG treatment for 6 h starting at 48 hpf; lateral views. Area A is situated outside the AGM region and area B represents the AGM region (outlined). Red bar marks approximate size of the dorsal aorta and blue bar that of the posterior cardinal vein. *n*=10 embryos from three different clutches. Scale bar, 100 μm. (**g**–**j**) Quantification of macrophage mobilization from the AGM based on *mfap4* WISH experiments, showing the macrophage absolute number in area A, macrophage absolute number in area B, ratio of macrophage number in area A to macrophage number in area B and total macrophage number. (**k**) Maximal intensity projections of confocal z-stacks of *Tg(kdrl:EGFP);Tg(mpeg:mCherry)* WT and *hif-1α*^*−/−*^embryos at 54 hpf. (**l**) Quantification of macrophage mobilization from the AGM of 54 hpf *Tg(kdrl:EGFP);Tg(mpeg:mCherry)* WT siblings and *hif-1α*^*−/−*^embryos. (**m**) Mosaic vessels from transplantation of WT *Tg(kdrl:ras-mCherry)* donor cells into *hif-1α*^*−/−*^ *Tg(kdrl:EGFP)* host blastulae. (**n**) Quantification of macrophage mobilization from the AGM in control *Tg(kdrl:EGFP);Tg(mpeg:mCherry) hif-1α*^*−/−*^and WT EC-transplanted *hif-1α*^*−/−*^embryos at 54 hpf. Arrowheads point to macrophages in the AGM. Bars represent mean±s.e.m., *n*=10 embryos from three different clutches (*P<0.05; **P<0.01; ***P<0.001; ****P<0.0001; NS, no significant changes observed; *t*-test). Scale bars, 50 μm.

It has been shown that *Hif-1α* plays an important role in regulating myeloid cell metabolism and function^22^,^31^. Interestingly, we found by qPCR analysis that macrophage genes such as *lcp1* and *mfap4* were down-regulated in *hif-1α*^*−/−*^ compared to WT siblings (Supplementary Fig. 2d,e). To investigate whether macrophage behaviour was impaired in *hif-1α* mutants, we performed whole-mount *in situ* hybridization (WISH) for *mfap4*, a macrophage marker gene. In WT siblings, in normoxic conditions as well as after hypoxia chamber incubation or DMOG treatment, macrophages were found both outside (area A) and inside (area B) the AGM region^32^ (Fig. 3a–c). In contrast, in *hif-1α*^*−/−*^ in normoxic conditions, most macrophages remained inside the AGM (Fig. 3d); and similarly on hypoxia chamber incubation or DMOG treatment little macrophage mobilization from the AGM was observed in *hif-1α*^*−/−*^ (Fig. 3e,f,i). Accordingly, WT siblings presented a significantly (*t*-test) higher number of macrophages outside the AGM compared to *hif-1α*^*−/−*^ (Fig. 3g), whereas the opposite was observed in *hif-1α*^*−/−*^ where most macrophages remained inside the AGM (Fig. 3h). However, total macrophage numbers were similar in WT and *hif-1α*^*−/−*^ (Fig. 3j). Altogether, these results suggest that the differences in macrophage distribution between WT and *hif-1α*^*−/−*^ embryos are most likely due to defects in macrophage localization rather than survival and/or proliferation rate. Similarly, co-injection of *hif-1aa* and *hif-1ab* morpholinos (MOs) in animals, hereafter referred to as *hif-1α* morphants, led to impaired macrophage mobilization from the AGM in normoxia and after hypoxic stress or DMOG treatment (Supplementary Fig. 5a–g).

To investigate whether other leucocytes are also affected in *hif-1α*^*−/−*^, we performed WISH for *mpx*, which is specifically expressed in neutrophils. We did not observe differences in neutrophil localization in normoxia or after hypoxia chamber incubation or DMOG treatment between WT and *hif-1α*^*−/−*^ (Supplementary Fig. 6a–f), although we did notice differences in neutrophil numbers (Supplementary Fig. 6g).

To gain further insight into the consequences that lack of *hif-1α* function has in macrophage mobilization and behaviour, we crossed *hif-1α* mutants into the macrophage reporter line *Tg(mpeg1:mCherry-F)*^*ump2*^, hereafter *Tg(mpeg:mCherry)*. By confocal imaging, we detected a significant (*t*-test) reduction in macrophage mobilization from the AGM in *hif-1α* mutants compared to WT siblings (Fig. 3k,l), confirming our previous observations. This result was further validated in *hif-1α* morphants (Supplementary Fig. 5h). Lastly, to test whether *hif-1α* in ECs influences macrophage mobilization from the AGM, we generated genetically mosaic DAs and posterior cardinal veins by transplanting at mid-blastula stages WT *Tg(kdrl:Hsa.HRAS-mCherry)*^*s896*^, hereafter *Tg(kdrl:ras-mCherry),* cells into *hif-1α*^*−/−*^ *Tg(kdrl:EGFP)* animals. Our observations revealed that the presence of WT ECs in these vessels did not rescue the macrophage mobilization phenotype (Fig. 3m,n), suggesting that the macrophage mobilization defects are independent of the expression of *hif-1α* in ECs. We also mosaically expressed under the control of the *mpeg* promoter WT Hif-1ab in *hif-1α*^*−/−*^ or a dominant-negative version of Hif-1ab (dn-Hif-1ab)^33^ in WT embryos, tagging these transgenes with TagBFP expression (Supplementary Fig. 7a). *Tg(mpeg:*mCherry)^+^ and TagBFP^−^ macrophages exhibited active protrusions of filopodia and lamellipodia (Supplementary Fig. 7b). In contrast, all the *WT-hif-1ab-* and *dn-hif-1ab-*expressing macrophages, positive for both *Tg(mpeg:*mCherry) and TagBFP expression, lacked cytoplasmic extensions (Supplementary Fig. 7c,d), indicating that expression of these transgenes under the *mpeg* promoter seems to be toxic to macrophages, and thus one will need to generate additional tools to look at the cell-autonomous function of Hif-1α in macrophages. Altogether, our data show that *hif-1α* function in non-ECs, and possibly in macrophages themselves, is required for their mobilization from the AGM.

### Macrophage ablation recapitulates the *hif-1α* ^*−/−*^phenotypes

**Figure 4:**
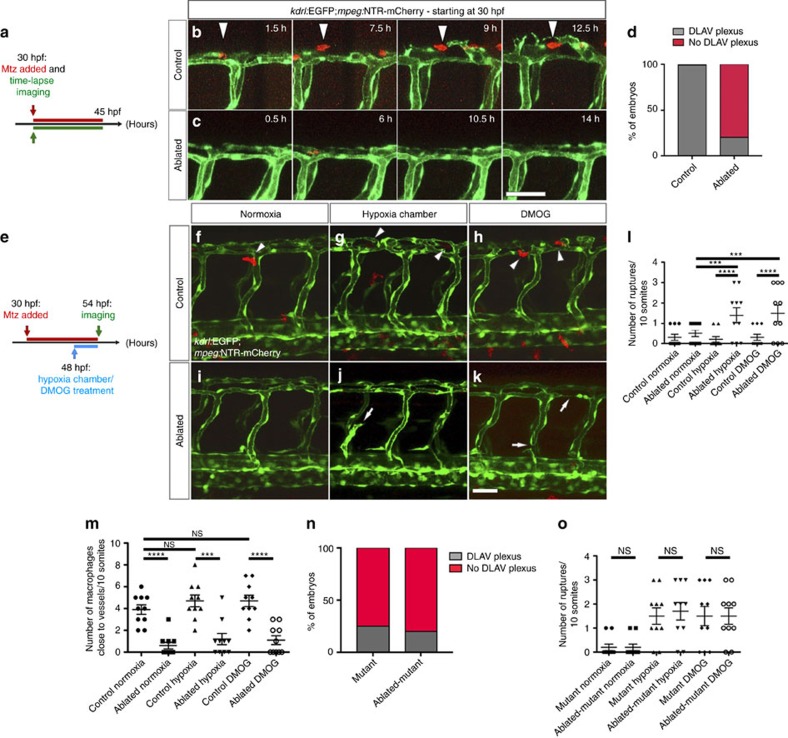
Macrophage ablation leads to the absence of DLAV plexus formation as well as hypoxia-induced vascular disconnections. (**a**) Schematic representation of the experiment shown in **b**–**d**. (**b**,**c**) Maximal intensity projections of time-lapse confocal images of control *Tg(kdrl:EGFP);Tg(mpeg:NTR-mCherry)* and macrophage-ablated embryos in normoxia starting at 30 hpf. Arrowheads point to a macrophage co-localizing with EC sprouts. (**d**) Quantification of embryos showing normal (grey) or abnormal/absent (red) DLAV plexus formation in 54 hpf control and macrophage-ablated embryos. (**e**) Schematic representation of the experiment shown in **f**–**m**. (**f**–**k**) Maximal intensity projections of confocal z-stacks of 54 hpf control *Tg(kdrl:EGFP);Tg(mpeg:NTR-mCherry)* and macrophage-ablated embryos in normoxia, after hypoxia chamber or DMOG treatment for 6 h starting at 48 hpf. All images represent lateral views. Arrowheads point to macrophages in proximity to blood vessels, and arrows to vessel ruptures. (**l**) Quantification of blood vessel ruptures in a 10 somite-long trunk area in 54 hpf control and macrophage-ablated embryos in normoxia, after hypoxia chamber or DMOG treatment for 6 h starting at 48 hpf. (**m**) Quantification of macrophages in proximity to blood vessels in a 10 somite-long trunk area in 54 hpf control and macrophage-ablated embryos in normoxia, after hypoxia chamber or DMOG treatment for 6 h starting at 48 hpf. *n*=10 embryos for each condition. (**n**) Quantification of embryos showing normal (grey) or abnormal/absent (red) DLAV plexus formation in 54 hpf control and macrophage-ablated *hif-1α*^*−/−*^embryos. (**o**) Quantification of blood vessel ruptures in a 10 somite-long trunk area in 54 hpf control and macrophage-ablated *hif-1α*^*−/−*^embryos in normoxia, after hypoxia chamber or DMOG treatment for 6 h starting at 48 hpf. Bars represent mean±s.e.m., *n*=10 embryos from three different clutches, (***P<0.001; ****P<0.0001; NS, no significant changes observed; *t*-test). Scale bars, 50 μm.

**Figure 5:**
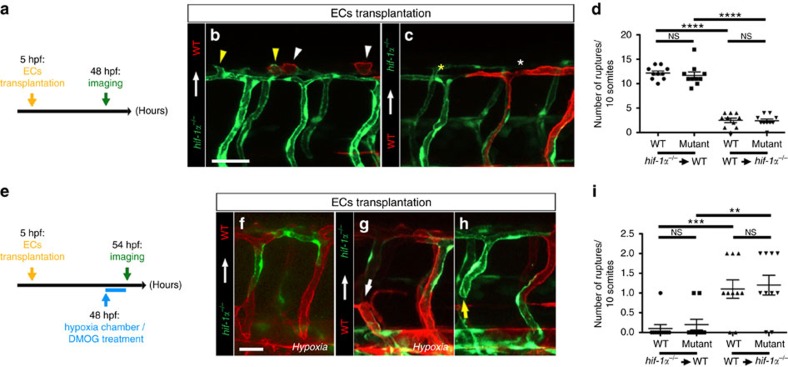
*hif-1α* does not play a cell-autonomous role in ECs during DLAV plexus formation or during blood vessel repair after hypoxia. (**a**) Schematic representation of the experiment shown in **b**–**d**. (**b**,**c**) Transplantation of *hif-1α*^*−/−*^ *Tg(kdrl:EGFP)* donor cells into WT *Tg(kdrl:ras-mCherry)* host blastulae and of WT *Tg(kdrl:ras-mCherry)* donor cells into *hif-1α*^*−/−*^ *Tg(kdrl:EGFP)* host blastulae shown at 48 hpf; white arrowheads point to sprouts from WT DLAV ECs, yellow arrowheads point to sprouts from *hif-1α*^*−/−*^ DLAV ECs, white asterisk indicates missing sprout from a WT EC and yellow asterisk indicates missing sprout from a *hif-1α*^*−/−*^ EC. (**d**) Quantification of DLAV sprouts in a 10 somite-long trunk area from WT and *hif-1α*^*−/−*^ DLAV ECs in the two different EC transplantation conditions at 48 hpf. (**e**) Schematic representation of the experiment shown in **f**–**i**. (**f**–**h**) Transplantation of *hif-1α*^*−/−*^ *Tg(kdrl:EGFP)* donor cells into WT *Tg(kdrl:ras-mCherry)* host blastulae, and of WT *Tg(kdrl:ras-mCherry)* donor cells into *hif-1α*^*−/−*^ *Tg(kdrl:EGFP)* host blastulae shown after hypoxia chamber or DMOG treatment for 6 h starting at 48 hpf; white arrow points to vessel rupture affecting WT EC, and yellow arrow points to vessel rupture affecting *hif-1α*^*−/−*^ EC. (**i**) Quantification of blood vessel ruptures in a 10 somite-long trunk area affecting WT and *hif-1α*^*−/−*^ ECs in the two different EC transplantation conditions at 54 hpf after hypoxia chamber or DMOG treatment for 6 h starting at 48 hpf. Bars represent mean±s.e.m., *n*=10 embryos from three different clutches, (**P<0.01; ***P<0.001; ****P<0.0001; NS, no significant changes observed; *t*-test). Scale bars, 50 μm.

To investigate whether macrophages are involved in the vascular defects observed in *hif-1α* mutants, we took advantage of the well-established genetic cell ablation method that uses the bacterial enzyme nitroreductase (NTR) to induce apoptotic cell death on administration of its substrate prodrug, metronidazole (Mtz)^34^,^35^,^36^. Here, we used the *Tg(mpeg1:Gal4-VP16)*^*gl24*^*;Tg(UAS-E1b:NTR-mCherry)*^*c264*^ lines, hereafter *Tg(mpeg:NTR-mCherry)*, to specifically ablate macrophages in a temporally controlled manner. First, to address whether DLAV angiogenesis was affected by macrophage depletion, we carried out time-lapse confocal imaging starting at 30 hpf of both control (DMSO-treated) and macrophage-ablated embryos (Fig. 4a). Interestingly, macrophages were observed to be closely associated with DLAV plexus formation, co-localizing with several sprouts (Fig. 4b,d and Supplementary Movie 1). Notably, macrophage-ablated embryos exhibited a complete absence of DLAV formation and sprouting (Fig. 4c,d and Supplementary Movie 2), reminiscent of the *hif-1α*^*−/−*^phenotype (Fig. 2e–g,l–n). To investigate whether macrophages might also play a role in vessel ruptures observed in *hif-1α*^*−/−*^, we combined the ablation treatment starting at 30 hpf with incubation for 6 h in the hypoxia chamber or DMOG starting at 48 hpf, followed by confocal imaging at 54 hpf (Fig. 4e). In control (DMSO-treated) embryos, macrophages were closely associated with blood vessels (Fig. 4f–h). In contrast, under hypoxic stress or DMOG treatment, macrophage-ablated embryos exhibited vascular ruptures (Fig. 4i–l), recapitulating the phenotype observed in *hif-1α* mutants (Fig. 2l–n). In addition, we quantified the number of macrophages in contact with ECs in the different conditions (Fig. 4f–k), and observed a significantly (*t*-test) higher number of macrophages in proximity to blood vessels in control (DMSO-treated) siblings than in the ablated embryos (Fig. 4m). Consistent with these observations, qPCR analysis revealed a down-regulation of blood vessel-macrophage interaction genes including *tek, spi1b* and *csf1a* in *hif-1α* mutants compared to WT siblings (Supplementary Fig. 2f–h). Next, we sought to determine whether *hif-1α* is acting cell-autonomously in ECs to regulate DLAV plexus formation and prevent vascular ruptures. First, we assessed whether *hif-1aa* and *hif-1ab* are expressed in ECs in WT embryos in normoxia or after incubation in the hypoxia chamber or DMOG treatment. These genes showed an overlapping expression pattern where *hif-1ab* expression levels were higher than those of *hif-1aa,* as previously described^33^,^37^. Both genes are expressed in the neural tube and pronephric region (Supplementary Fig. 8a,d), as previously reported^38^. After incubation in the hypoxia chamber or DMOG, only *hif-1ab* displayed increased expression levels, becoming detectable in the notochord and neuromasts (Supplementary Fig. 8b,c,e,f). Then, we performed macrophage ablation experiments in *Tg(mpeg:NTR-mCherry) hif-1α* mutants. Interestingly, macrophage ablation did not exacerbate the DLAV plexus (Fig. 4n) or blood vessel rupture (Fig. 4o) phenotypes in *hif-1α*^*−/−*^ consistent with the hypothesis that macrophages are central to the vascular defects observed in *hif-1α*^*−/−*^. To begin to test this model, we generated genetically mosaic DLAVs by cell transplantation at mid-blastula stages, and assessed the number of DLAV sprouts at 48 hpf (Fig. 5a). When *hif-1α*^*−/−*^ *Tg(kdrl:EGFP)* ECs were transplanted into WT *Tg(kdrl:ras-mCherry)* embryos, we observed that WT and *hif-1α*^*−/−*^ ECs exhibited a similar number of sprouts from the DLAVs (Fig. 5b,d). In contrast, when WT *Tg(kdrl:ras-mCherry)* ECs were transplanted into *hif-1α*^*−/−*^ *Tg(kdrl:EGFP)* embryos, we did not observe any DLAV EC sprouts (Fig. 5c,d), indicating that *hif-1α* is not required in ECs during DLAV plexus formation. In addition, we generated genetically mosaic ISVs and DLAVs by cell transplantation at mid-blastula stages and quantified the number of vascular ruptures after hypoxia (Fig. 5e). *hif-1α*^*−/−*^ *Tg(kdrl:EGFP)* ECs were transplanted into WT *Tg(kdrl:ras-mCherry)* embryos and no differences in the number of vessel ruptures were observed (Fig. 5f,i). When WT *Tg(kdrl:ras-mCherry)* ECs were transplanted into *hif-1α*^*−/−*^ *Tg(kdrl:EGFP)* embryos, we observed that blood vessel ruptures affected WT and mutant ECs equally, further supporting the model that vessel disconnections are not due to the lack of *hif-1α* in ECs (Fig. 5g–i). Altogether, our macrophage ablation and genetic mosaic data suggest that macrophages are required to support DLAV angiogenesis, as well as the repair of hypoxia-induced vessel ruptures.

### DLAV plexus formation is a macrophage-associated process

**Figure 6:**
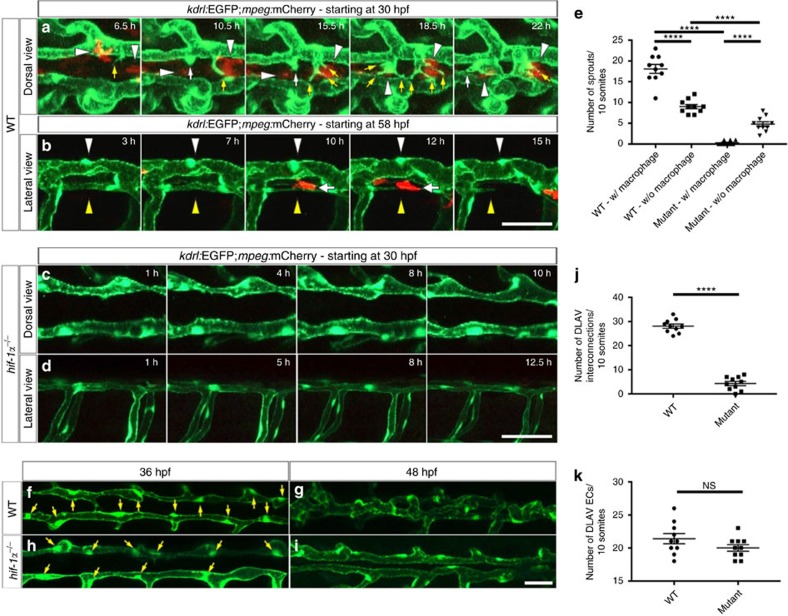
DLAV plexus formation is a macrophage-associated process and it is absent in *hif-1α* mutants. (**a**) Maximal intensity projections of time-lapse confocal images of *Tg(kdrl:EGFP);Tg(mpeg:mCherry)* WT sibling embryo starting at 30 hpf; dorsal views. White arrowheads point to macrophages, yellow arrows to endothelial filopodial extensions, and white arrows to blunt-ended endothelial protrusions. (**b**) Maximal intensity projections of time-lapse confocal images of *Tg(kdrl:EGFP);Tg(mpeg:mCherry)* WT sibling embryo starting at 58 hpf; lateral views. White arrowheads point to a segment of a stable and perfused DLAV vessel, yellow arrowheads point to a non-perfused and regressing DLAV vessel, and white arrows point to a macrophage in contact with the regressing vessel. (**c**,**d**) Maximal intensity projections of time-lapse confocal images of *Tg(kdrl:EGFP);Tg(mpeg:mCherry) hif-1α* mutants starting at 30 hpf in dorsal and lateral views. DLAV plexus formation fails to occur in *hif-1α* mutants. (**e**) Quantification of sprouts physically associated (w/) or not (w/o) to macrophages in WT and *hif-1α*^*−/−*^ embryos between 36 and 48 hpf. (**f**–**i**) Maximal intensity projections of confocal z-stacks of *Tg(kdrl:EGFP)* WT sibling and *hif-1α*^*−/−*^ embryos at 36 and 48 hpf; dorsal views. Yellow arrows point to nuclei. (**j**) Quantification of DLAV interconnections in WT and *hif-1α*^*−/−*^ embryos occurring between 36 and 48 hpf. (**k**) Quantification of EC numbers in the DLAVs in WT and *hif-1α*^*−/−*^ embryos at 36 hpf. Bars represent mean±s.e.m., *n*=10 embryos from three different clutches, (****P<0.0001; NS, no significant changes observed; *t*-test). Scale bars, 50 μm.

To further characterize DLAV plexus formation, we imaged WT animals by confocal microscopy. DLAV plexus morphogenesis is an evolutionarily conserved process driven by angiogenesis^28^,^39^,^40^. In zebrafish embryos, it begins with DLAV sprouting at around 36 hpf, followed by the formation of a transient plexus consisting of three longitudinal vessels by 60 hpf, which are finally rearranged into a single wide longitudinal vessel by 96 hpf^28^. By confocal imaging, we detected DLAV sprouts consisting of both filopodial and blunt-ended extensions that closely co-localized with macrophages (Figs 4b and 6a and Supplementary Movies 1 and 3). How this three vessel-plexus becomes remodelled into a single vessel has not yet been described. To study the dynamics of this process, we performed *in vivo* imaging between 60 and 72 hpf. Based on our confocal microscopy analysis, we observed that when one of the DLAV vessels was not perfused, it consequently underwent regression while the perfused vessel was preserved (Fig. 6b and Supplementary Movie 4). Interestingly, macrophages appear to contact unstable vessels just before their regression (Fig. 6b and Supplementary Movie 4). Next, we sought to determine whether *hif-1α* mutants exhibit any hints of sprouting from the DLAV. We imaged *hif-1α* mutants at 30 hpf, a time when DLAV sprouts begin to form in WT embryos, and did not observe any sprouts connecting the two longitudinal vessels, or any macrophages around the DLAV (Fig. 6c,d and Supplementary Movies 5 and 6). This result was confirmed in *hif-1α* morphants (Supplementary Fig. 9). Quantification of the number of DLAV sprouts closely associated with macrophages revealed that in WT siblings most of the DLAV sprouts co-localized with macrophages (Fig. 6e). In contrast, in *hif-1α*^*−/−*^embryos, we observed just a few sprouts from the DLAVs, and almost none of them were associated with macrophages (Fig. 6e). In addition, we determined how many DLAV interconnections were formed between 36 and 48 hpf (Fig. 6f–i), and observed a significant (*t*-test) reduction in *hif-1α*^*−/−*^(Fig. 6j). Importantly, we did not observe a significant (*t*-test) difference in the number of ECs forming the DLAV at 36 hpf (Fig. 6k). Thus, our confocal microscopy observations show that macrophages are closely associated with DLAV plexus formation, a process that fails to occur in *hif-1α* mutants and morphants.

### Macrophages do not assist in vessel repair in *hif-1α* mutants

**Figure 7:**
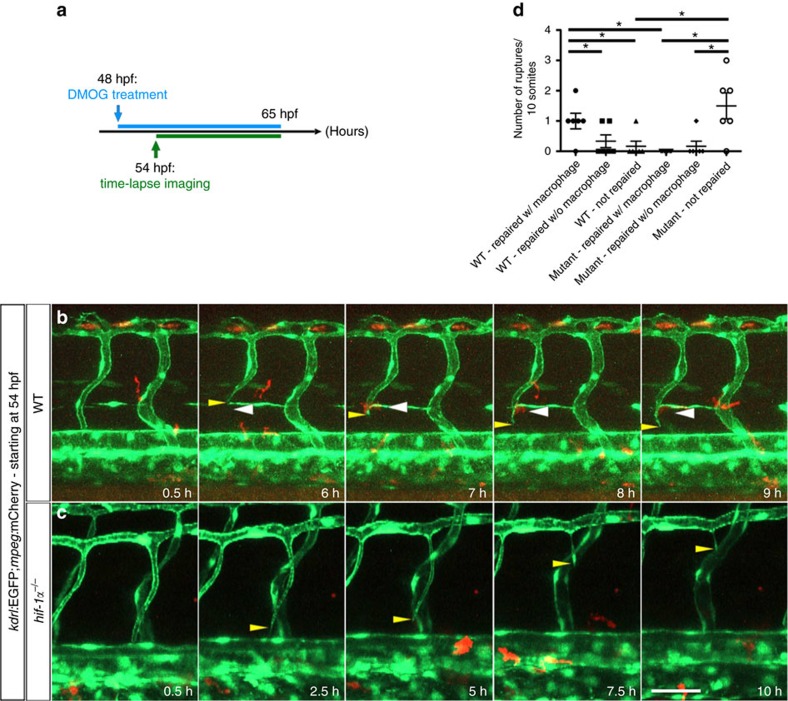
Macrophages do not appear to assist in vessel repair in *hif-1α* mutants. (**a**) Schematic representation of the experiment shown in **b**–**d**. (**b**) Maximal intensity projections of time-lapse confocal images of a *Tg(kdrl:EGFP);Tg(mpeg:mCherry)* WT sibling treated with DMOG starting at 48 hpf. Yellow arrowheads point to a vessel rupture and white arrowheads to macrophages. (**c**) Maximal intensity projections of time-lapse confocal images of a *Tg(kdrl:EGFP);Tg(mpeg:mCherry) hif-1α* mutant treated with DMOG starting at 48 hpf. Yellow arrowheads point to a vessel rupture and subsequent regression. (**d**) Quantification of blood vessel ruptures repaired in the presence (w/) or absence (w/o) of macrophages as well as those not repaired in WT and *hif-1α*^*−/−*^ embryos at 65 hpf. All images represent lateral views. Bars represent mean±s.e.m., *n*=6 embryos from three different clutches, (*P<0.05; *t*-test). Scale bar, 50 μm.

**Figure 8:**
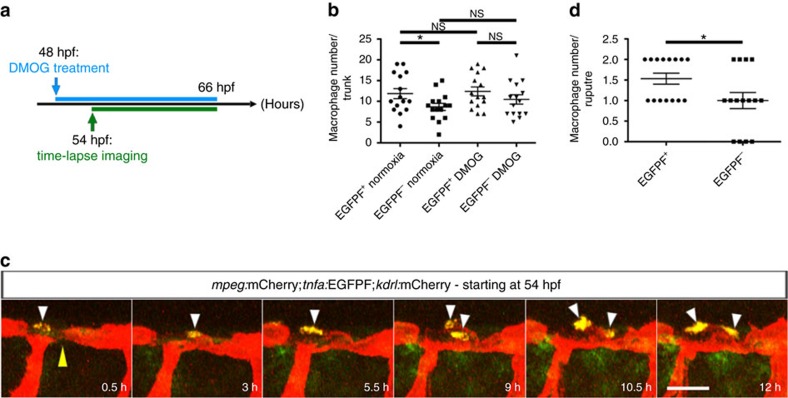
Most macrophages closely associated with unstable vessels are *tnfa:*EGFPF^+^. (**a**) Schematic representation of the experiment shown in **b**–**d**. (**b**) Quantification at 66 hpf of *tnfa:*EGFPF^+^ and *tnfa:*EGFPF^−^ macrophages in normoxia and after DMOG treatment for 18 h starting at 48 hpf. (**c**) Maximal intensity projections of time-lapse confocal images of *Tg(mpeg:mCherry);Tg(tnfa:EGFPF);Tg(kdrl:mCherry)* WT embryo treated with DMOG starting at 48 hpf; lateral views. Yellow arrowhead points to an unstable vessel, white arrowheads point to recruited macrophages. (**d**) Quantification at 66 hpf of *tnfa:*EGFPF^+^ and *tnfa:*EGFPF^−^ macrophages associated with 15 unstable vessels after DMOG treatment starting at 48 hpf. 38 macrophages were observed, 23 *tnfa:*EGFPF^+^ and 15 *tnfa:*EGFPF^−^. Bars represent mean±s.e.m., *n*=15 embryos from three different clutches, (*P<0.05; NS, no significant changes observed; *t*-test). Scale bar, 50 μm.

To determine whether macrophages are directly involved in vessel rupture resolution, we performed time-lapse imaging on DMOG-treated WT and *hif-1α* mutant embryos starting at 54 hpf (Fig. 7a). When a vessel rupture occurred in WT animals, several macrophages moved towards the rupture and closely interacted with it, thereby likely supporting vessel regeneration and reconnection with the main vessel (Fig. 7b and Supplementary Movie 7). Visual analysis using the *Tg(kdrl:EGFP);Tg(gata1:DsRed)* lines confirmed that repaired blood vessels in WT animals regained blood flow (Supplementary Fig. 10). In contrast, in *hif-1α* mutants, macrophages did not interact with the collapsed extremities of injured vessels and subsequently the break was not repaired, resulting in vessel disconnection from the DA (Fig. 7c and Supplementary Movie 8). Similar observations were made in *hif-1α* morphants (Supplementary Fig. 11). Quantification further confirmed that in WT siblings most of the ruptured vessels interacted with macrophages and were repaired, and that in *hif-1α* mutants most blood vessel ruptures did not interact with macrophages and were not repaired (Fig. 7d). To better characterize the molecular signature of macrophages involved in vessel repair, we used the recently published *Tg(tnfa:EGFP-F)*^*ump5*^ reporter line^41^, hereafter *Tg(tnfa:EGFPF*), and performed DMOG treatment. First, *Tg(tnfa:EGFPF*) embryos were treated with DMOG for 18 h starting at 48 hpf (Fig. 8a) to assess whether DMOG itself modulates *Tg(tnfa:EGFPF)* expression. Quantification of *tnfa:*EGFPF^+^ and *tnfa:*EGFPF^−^ macrophages showed that DMOG treatment did not affect the number of *tnfα*-expressing macrophages (Fig. 8b). Importantly, we subsequently observed that most macrophages in close association with unstable vessels were *tnfa:*EGFPF^+^ (Fig. 8c,d and Supplementary Movie 9). Together with our macrophage ablation data, these observations indicate that macrophages are required for vessel repair and that in the absence of *hif-1α* this repair does not take place. Moreover, most macrophages closely associated with vessel ruptures caused by DMOG treatment are *tnfa:*EGFPF positive.

## Discussion

**Figure 9:**
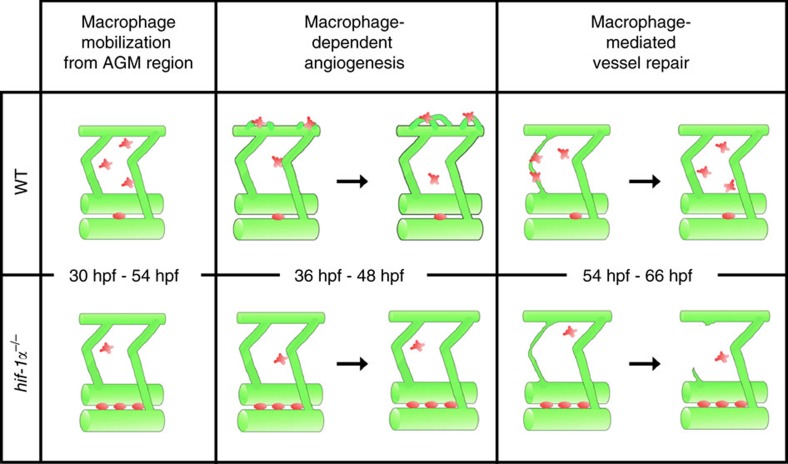
*hif-1α* modulates macrophage-endothelial interactions during blood vessel development and repair. *hif-1α* regulates macrophage interactions with ECs during blood vessel development and repair. At first, in *hif-1α* mutants, macrophages show reduced mobilization from the AGM region. In addition, DLAV plexus morphogenesis is a macrophage-dependent angiogenic process. *hif-1α* mutants and macrophage-ablated animals show no DLAV plexus formation. And, hypoxic conditions induce vessel ruptures: in WT embryos, macrophages appear to interact with the broken vessel, possibly supporting its reconnection to the main vessel. In *hif-1α* mutants and in macrophage-ablated animals, macrophages do not interact with the ruptured vessel, the damage is not repaired, and the ruptured vessel regresses. Vessels are shown in green, macrophages in red.

The presence of hypoxic areas and aberrant activation of the HIF pathway are hallmarks of many pathological tissues including solid tumours, wounds, as well as sites of infection and inflammation. Macrophages are able to adapt to hypoxia and subsets of adult macrophages have been implicated in these and other pathological settings. Notably, the first macrophages differentiate in the embryo and persist into adulthood as tissue macrophages^42^. However, the role of these early tissue macrophages in embryonic angiogenesis has not yet been extensively explored. In particular, how Hif1-α modulates macrophage function in vascular development has not been reported. Here we identify a role for *hif-1α* in modulating the interactions between macrophages and ECs during macrophage mobilization from the AGM, developmental angiogenesis and vessel repair after hypoxic conditions (Fig. 9).

*Hif-1α* has been described to be a key regulator of myeloid cell function at different levels, therefore being pivotal to an efficient and coordinated immune response^43^. Recently, studies in adult mice have shown that *Hif-1α* is necessary for HSC mobilization from the bone marrow to the blood and spleen^44^. Here, we report that *hif-1α* is required for macrophage mobilization from the AGM during zebrafish embryonic development. In three different experimental conditions (normoxia, hypoxia chamber incubation and DMOG treatment), we observed a significant (*t*-test) difference in macrophage mobilization between WT siblings and *hif-1α* mutants. Possible defects in cytoskeleton rearrangement in *hif-1α*^*−/−*^ macrophages may affect macrophage mobilization as suggested by a recent study reporting that Cdc42, involved in lamellipodia formation, and Rac1, important for stress fibre formation, are transcriptional targets of the HIF-1 pathway in macrophages^45^. Consistent with this model, myeloid-specific deletion of *Hif-1α* in mice causes abnormalities in motility, adhesion and invasiveness of peritoneal macrophages^22^. Moreover, it has been described that the expression of chemokine receptors, including CXCR4, in myeloid cells is regulated by the VHL-HIF axis^46^,^47^, giving another possible explanation for the lack of macrophage mobilization in *hif-1α*^*−/−*^ embryos.

Macrophages can stimulate endothelial proliferation and angiogenesis by secreting pro-angiogenic factors^1^,^48^ and stabilizing tip cell fusion, thereby increasing vascular complexity^11^; however, up to now it has not been reported that macrophages are required for developmental angiogenesis. DLAV plexus morphogenesis, an angiogenic process, begins at around 36 hpf in zebrafish, and it has been described to be a VEGF-dependent process^28^. In this study, we show that macrophage depletion completely abrogates DLAV plexus formation, recapitulating the *hif-1α*^*−/−*^ phenotype. Moreover, we observe that macrophages are closely associated with DLAV sprouts in WT siblings, being required in the initiation of EC sprouting behaviour. Hence, we provide direct evidence that macrophages are required for developmental angiogenesis in a specific vascular setting. On the other hand, ISV formation appeared unaffected in *hif-1α*^*−/−*^ as well as in macrophage-ablated animals. We thus speculate that at least two different types of angiogenesis can occur in zebrafish: a macrophage-independent angiogenesis, mainly driven by the VEGF-Notch cross-talk^49^, and a macrophage-dependent angiogenesis, whereby macrophages guide EC sprouting, possibly by secreting growth factors and/or by providing physical support. Moreover, DLAV angiogenesis leads to a transient pattern at ∼58 hpf, and subsequently the three axial vessels constituting the DLAV plexus rearrange into a single vessel by 120 hpf. According to our observations, this transition from a three- to one-vessel plexus happens through vessel regression whereby macrophages, by physical interaction with regressing vessels, may guide the pruning event. In this theme, previous studies in mice have reported the role of macrophages as negative regulators of vascular viability and branching^50^,^51^. What are the molecular cues that induce these two different types of macrophage behaviour, that is, initially promoting new vessel formation and subsequently inducing vessel regression during DLAV morphogenesis? Answering this question will improve our understanding of how macrophages can both positively and negatively regulate angiogenesis, thereby helping studies in cancer biology, where tumour-associated macrophages can also play a dual role in tumour growth^52^.

Liu *et al*.^53^ reported that macrophages support the repair of brain vascular ruptures through physical interactions. Interestingly, in their transcriptional analysis they detected a strong up-regulation of the Hif-1 pathway in the macrophages involved in vessel repair. In our study, we focused on *hif-1α* function during vascular development. Our observations point to a key role for *hif-1α* in modulating a macrophage function that is instrumental for vessel repair under hypoxic conditions. Macrophages are subdivided into two main classes: M1 that express *Tnfα* and are considered pro-inflammatory and anti-angiogenic, and M2, which are anti-inflammatory and pro-angiogenic. The classification of macrophages into these two subtypes is an oversimplification, as it excludes the intermediate states between these extremes. As we observed, macrophages that interact and support injured vessels in hypoxia-treated embryos are mostly *tnfa:*EGFPF^+^. This observation supports the notion that developmental macrophages are able to respond to their microenvironment and provide pleiotropic functions^54^, thereby going beyond the classical binary categorization of macrophages. Moreover, Liu *et al*.^53^ induced blood vessel damage by laser ablation and reported that macrophages repair these vascular ruptures through mechanical traction whereby they mediate vessel ligation by pulling EC ends together. In our model, blood vessel ruptures were induced by hypoxic conditions and we observed macrophages migrating towards the site of vascular damage where they supported ECs until they extended and reconnected to the main vessel. Our results are thus more similar to those reported by Fantin *et al*. who suggested that tissue macrophages act as chaperones in vascular anastomosis^9^. Moreover, several publications have described how hypoxia strongly influences macrophage mobilization towards tumours or inflammatory environments^46^,^55^,^56^. Intriguingly, clinical studies have suggested that thin walled blood vessels in combination with hypoxia-induced haemorrhage could be a major factor in the pathology that follows the birth of severely hypoxic fetuses, or which arises in neonates when resuscitation is difficult and prolonged^57^. Therefore, understanding whether macrophages have a role in repairing hypoxia-induced vessel ruptures could have significance in developing novel strategies to improve the outcome of these patients.

Altogether, in this study we provide genetic evidence for an important role for Hif-1α signalling in regulating the crosstalk between macrophages and ECs. We found that *hif-1α* regulates macrophage activity, unveiling new roles for macrophages during cardiovascular system development in both normoxic and hypoxic conditions. We show that *hif-1α* is required for macrophage mobilization from the AGM, developmental angiogenesis and vessel repair after hypoxic conditions. Data from our mosaic analyses indicate that Hif-1α is not required in ECs during macrophage mobilization from the AGM, or during DLAV plexus formation or vessel repair, and given previous reports about cell-autonomous functions of HIF-1α in macrophages^22^,^43^,^58^, it is reasonable to hypothesize that the function of Hif-1α during macrophage mobilization from the AGM is also, at least in part, cell-autonomous. Macrophage ablation leads to similar defects in developmental angiogenesis and vessel repair as those observed in *hif-1α* mutants. Whether *hif-1α* also functions cell-autonomously in macrophages during these processes will require the use of genetic tools that allow precise spatio-temporal inactivation of *hif-1α*, an approach currently feasible in mouse but not yet in zebrafish. Overall, our data provide new insights into the interactions between myeloid cells and ECs in both normoxic and hypoxic conditions, likely impacting on our understanding of pathological conditions where macrophages and ECs have to deal with changes in O_2_ levels.

## Methods

No statistical methods were used to predetermine sample size. The experiments were not randomized. The investigators were not blinded to allocation during experiments or outcome assessment, except for the data shown in Figs 2 and  3a–j.

### Zebrafish handling

Zebrafish husbandry was performed under standard conditions in accordance with institutional (MPG) and national ethical and animal welfare guidelines.

Zebrafish were maintained and embryos were obtained and raised under standard conditions^59^. *Tg(kdrl:EGFP)*^*s843*^ (ref. 60), *Tg(mpeg1:mCherry-F)*^*ump2*^ (ref. 61), *Tg(mpeg1:Gal4-VP16)*^*gl24*^ (ref. 62), *Tg(UAS-E1b:NTR-mCherry)*^*c264*^ (ref. 63), *Tg(kdrl:Hsa.HRAS-mCherry)*^*s896*^ (ref. 64), *Tg(gata1a:DsRed)*^*sd2*^ (ref. 65), *Tg(tnfa:EGFP-F)*^*ump5*^ (ref. 41) fish were used in this study.

### Generation of *hif-1aa* and *hif-1ab* mutants

pT7-gRNA and pT3TS-nlsCas9nls vectors were purchased from Addgene (http://www.addgene.org/46759/ and http://www.addgene.org/46757/). A gRNA was designed to target exon 6 of *hif-1aa* (Supplementary Table 1) using CRISPR design (http://crispr.mit.edu/) (Zhang laboratory). Oligonucleotides were annealed in a thermo block at 95 °C for 5 min followed by a slow cooling at room temperature and cloned into the gRNA plasmid between BsmbI sites. All constructs were verified by sequencing. To make gRNA, the template DNA was linearized by BamHI digestion and purified using a QIAprep column (Quiagen). gRNA was generated by *in vitro* transcription using a T7 RNA polymerase MEGA short script T7 kit (Life Technologies). After *in vitro* transcription, the gRNA (approximately 100 nucleotides long) was purified using RNA Clean and Concentrator kit (Zymo Research). To make nlsCas9nls RNA, the template DNA was linearized by XbaI digestion and purified using a QIAprep column (Qiagen). Capped nlsCas9nls RNA was synthesized using a mMESSAGE mMACHINE T3 kit (Life Technologies) and purified using an RNA Clean and Concentrator kit (Zymo Research). nlsCas9nls mRNA (100 pg) and gRNA (50 pg) were co-injected in the cell at the one-cell stage. Mutant alleles were identified by high-resolution melt analysis^66^ of PCR products generated with the following primers: *hif-1aa* forward: 5′-GGTGCTCATCTGCGAGTCTA-3′, *hif-1aa* reverse: 5′-GCTGAGGAACGTTCTGGAAT-3′.

A TALEN targeting exon 6 of *hif-1ab* was designed and cloned using Golden Gate assembly into the pCS2TAL3RR or pCS2TAL3DD expression vectors^25^. The TALEN was composed of the following TAL effector domains RVDs: NH NG NH HD NG HD NI NG HD NG NH NG NH NI NH HD HD HD NI NG and NH NG HD HD NI NI NG NH NH HD NI HD HD NG HD NH NI NG NH NG. One-cell stage embryos were injected with 50 pg total TALEN capped messenger RNA synthesized using the Sp6 mMESSAGE mMACHINE kit (Life Technologies) and purified using RNA clean and concentrator (Zymo Research). Mutant alleles were identified by high-resolution melt analysis^66^ of PCR products generated with the following primers: *hif-1ab* forward: 5′-CTCATCTGTGAGCCCATTCC-3′, *hif-1ab* reverse: 5′-GCTGAGGAAGGTCTTGCTGT-3′.

### Hypoxia chamber and dimethyloxalylglycine treatments

The hypoxia chamber was flushed with nitrogen gas to reach 5% O_2_ concentration (C-Chamber, ProOX 110, ProCO_2_ from Biospherix) at 28 °C. 50-mm Petri dishes containing 4 ml of embryo water were pre-equilibrated in the hypoxia chamber overnight. Treatments started at 24 or 48 hpf for 6 h, or at 58 hpf for 14 h.

Embryos were treated with 100 μM DMOG (Sigma-Aldrich) or with 0.5% dimethylsulfoxide (DMSO; Sigma-Aldrich) in 4 ml embryo medium at 28 °C in 50-mm Petri dishes. Treatments started at 48 hpf for 6 h. When performing time-lapse imaging, the treatment started at 48 hpf for up to 18 h.

### Imaging

Confocal images were acquired using LSM 700, LSM780, LSM800 confocal laser scanning microscopes (Zeiss) and high-end stereoscopic microscopes (Nikon SMZ25) after embryo anaesthesia with a low dose of tricaine and immobilization in 1% low-melting agarose in glass-bottom Petri dishes (MatTek Corporation, Ashland, MA, USA). Time-lapse images were recorded every 30 min for a maximum period of around 16 h. The microscope stage was enclosed in a temperature-controlled chamber, and samples were kept at 28 °C. Vessel integrity and permeability were analysed using microangiography. Rhodamine-dextran 150 kDa (Sigma-Aldrich) was injected into the common cardinal vein at ∼53 hpf and imaged after 30 min.

### Quantitative PCR analysis

qPCR was performed on cDNA obtained from 48 hpf WT sibling and mutant RNA extracted using TRIZOL (Life Technologies) followed by washes with chloroform to isolate the RNA and with isopropanol to precipitate it^67^,^68^. When qPCR was performed to validate microarray data, cDNA was obtained from 50 hpf WT sibling and mutant RNA extracted using TRIZOL (Life Technologies) after hypoxia chamber treatment (2 h in 3% O_2_). DNase treatment was performed for 30 min at 37 °C (Promega) followed by RNA purification with RNA Clean and Concentrator kit (Zymo Research). cDNA was synthesized using SuperScript III RT (Invitrogen) starting from 500 μg of RNA. A Bio-Rad Real-Time PCR System was used for qPCR experiments, and gene expressions were normalized relative to that of the zebrafish *18S ribosomal RNA* gene. All reactions were performed in three technical replicates, and the results represent three independent biological samples (30 embryos pooled for each sample). qPCR primers: *hif-1aa* forward: 5′-AGCCGCCACACTTTAGACAT-3′ and *hif-1aa* reverse: 5′-CCTCTGGATCAAAACCCAAG-3′; *hif-1ab* forward: 5′-GCCACACTCTGGACATGAAG-3′ and *hif-1ab* reverse: 5′-TCAAGAGGTCATCTGGCTCA-3′; *18S ribosomal RNA* forward: 5′-TCGCTAGTTGGCATCGTTTATG-3′ and *18S ribosomal RNA* reverse: 5′-CGGAGGTTCGAAGACGATCA-3′; *phd3* forward: 5′-CCTGGAAATGGAGCTGGATA-3′ and *phd3* reverse: 5′-CCGGTCAAATAAAGGCTCAA-3′; *hbbe3* forward: 5′-CTCAGCGAGCTTCACTCAGA-3′ and *hbbe3* reverse: 5′-GACAGGAACTTCTGCCAAGC-3′; *pfkfb3* forward: 5′-GCAAACCCTCCAACAGTGAT-3′ and *pfkfb3* reverse: 5′-GTTTCACTGCTTCACGACGA-3′; *lcp1* forward: 5′-CGGAAGGCCATCAATAAGAA-3′ and *lcp1* reverse: 5′-CCTTCTCCAGAGCCTTGTTG-3′; *mfap4* forward: 5′-TGCTCTCAGATGGGAAAGATG-3′ and *mfap4* reverse: 5′-GCCAGTATTCTCCCTCCACA-3′; *tek* forward: 5′-AGCTCCAGGAACACTGAGGA-3′ and *tek* reverse 5′-ATGTGGAGCTGCTGTGTCTG-3′; *spi1b* forward: 5′-ATGTGGAGTCCAGCCATTTC-3′ and *spi1b* reverse: 5′-TGGACGTTGTGAGGGTAACA-3′; *csf1a* forward: 5′-AAAAACCAGCTGCAAAATGG-3′ and *csf1a* reverse: 5′-ATTGTCGGAATCCTTTGCAT-3′.

### Microarray analysis

RNA was extracted using TRIZOL (Life Technologies) from 30 *hif-1α* mutants and 30 WT siblings. Two different conditions were analysed (one sample for each condition): 50 hpf WT and mutants in normoxia, 50 hpf WT and mutants after hypoxia chamber treatment (2 h in 3% O_2_). DNase treatment was performed for 30 min at 37 °C (Promega). After purification with RNA Clean and Concentrator kit (Zymo Research), the microarray analysis was performed by Oaklabs (Hennigsdorf, Germany). A 8 × 60 K zebrafish expression array (XS-5090; Agilent 60-mer SurePrint technology) analysis was performed according to manufacturer's protocol.

### Whole-mount *in situ* hybridization

WISH probes were generated using the following primers: *mfap4*-ISH forward: 5′-TGTTCTTGGCGACGCTTCT-3′ and *mfap4*-ISH reverse: 5′-TAATACGACTCACTATAGGGTGATGGGTGGCATCTTCTC-3′; *mpx*-ISH forward: 5′-ATTAACCCTCACTAAAGGGAGTATCGAACTGCCAGCGGTGTCT-3′ and *mpx*-ISH reverse: 5′-TAATACGACTCACTATAGGG ACGGTCTCCTCTCTGTAGGCTCA-3′; *hif-1aa*-ISH forward: 5′-TCAGAGAAATGCTGGCACAC-3′ and *hif-1aa*-ISH reverse: 5′-TAATACGACTCACTATAGAACCCACTCCCTGTGTCTTG-3′; *hif-1ab*-ISH forward: 5′-CCAGTGGAACCAGACATCAG-3′ and *hif-1ab*-ISH reverse: 5′-TAATACGACTCACTATAGGACTTGGTCCAGAGCACGC-3′. T7 was used for transcription and digoxigenin labelling of probes. For whole-mount *in situ* hybridization, embryos were fixed in 4% paraformaldehyde overnight at 4 °C and subsequently dehydrated in methanol and stored at −20 °C until needed. In the first day, embryos were rehydrated to PBS/0.1% Tween-20 and then digested in 10 μg ml^−1^ proteinase K (Roche) followed by fixation in 4% PFA. Embryos were pre-incubated with hybridization buffer at 70 °C for 3 h and then incubated with DIG-labelled RNA antisense probes at 70 °C overnight. The next day, after washing, the embryos were incubated with alkaline phosphatase-conjugated anti-digoxigenin antibody (Roche) at 4 °C overnight. The last day, after washing, the signal was visualized with NBT-BCIP staining solution (Roche)^68^.

### Morpholinos

ATG MOs against *hif-1aa* (5′-TTTTCCCAGGTGCGACTGCCTCCAT-3′)^69^, *hif-1ab* (5′-ACCCTACAAAAGAAAGAAGGAGAGC-3′)^70^ and control MO (5′-CCTCTTACCTCAGTTACAATTTATA-3′) were purchased from Gene Tools (Eugene, OR) and injected at the one-cell stage at 0.5 ng/embryo for each MO. This dose was determined as optimal (no overt toxic effects observed) by titration and injection of *hif-1aa* and *hif-1ab* MOs singly and together; when co-injected, *hif-1aa* and *hif-1ab* MOs recapitulated the morphological phenotypes observed in *hif-1α* mutants.

### Cell transplantations

*Tg(kdrl:EGFP)* and *Tg(kdrl:Hsa.HRAS-mCherry)* embryos were dechorionated using pronase (1 mg ml^−1^) for 5 min at 28 °C in 1/3 Ringer solution supplemented with penicillin (50 U ml^−1^)/streptomycin (50 μg ml^−1^) before being incubated in agarose-coated dishes in the same medium. Cells were taken from donor embryos at mid-blastula stages and transplanted along the blastoderm margin of age-matched host embryos which were subsequently grown at 28.5 °C until the indicated stages. The contribution of transplanted cells was assessed using a Nikon SMZ25 stereomicroscope, and EC position within mosaic vessels was determined using confocal microscopy.

### Transgenic macrophage expression

A dominant-negative form of *hif-1ab (dn-hif-1ab)* was generated using primers amplifying DNA corresponding to amino acids 1-330 of human HIF-1α (ref. 33). WT form of *hif-1ab* was cloned using the following primers: *hif-1ab* forward: 5′-ATGGATACTGGAGTTGTCACT-3′ and *hif-1ab* reverse: 5′-TCAGTTGACTTGGTCCAGAGC-3′. *mpeg1* promoter was generated from genomic DNA using the following Gateway primers: *mpeg1*-AttB1R: 5′-GGGGACTGCTTTTTTGTACAAACTTGTTTTGCTGTCTCCTGCAC-3′; *mpeg1*-attB4: 5′-GGGGACAACTTTGTATAGAAAAGTTGTTGGAGCACATCTGAC-3′ (ref. 62). Expressing cells were visualized by fusing the *dn-hif-1ab* or *WT-hif-1ab* coding sequence to TagBFP (Evrogen) by a ‘self-cleaving' viral 2 A peptide sequence. Transient mosaic overexpression was obtained by co-injecting 50 pg of Tol2 transposase mRNA and 75 pg of the pTol2-*mpeg:TagBFP-2A-dn-hif-1ab* or pTol2-*mpeg:TagBFP-2A-WT-hif-1ab* plasmid DNA.

### Metronidazole treatment

Mtz (Sigma, M3761) was used at a 2 mM concentration dissolved in egg water containing 0.5% DMSO for all of the cell ablation experiments conducted in this study. Before treatment with 2 mM Mtz or control 0.5% DMSO, embryos were manually dechorionated with forceps and then incubated with freshly prepared 2 mM Mtz or 0.5% DMSO in egg water. To wash away the Mtz, fish embryos/larvae were washed twice in dishes containing egg water. About 5% of the treated animals developed severe gross anatomical abnormalities. Only animals that appeared morphologically unaffected were analysed.

### Quantification and statistical analysis

To quantify DLAV plexus formation, we counted the number of embryos that showed a complete lack of DLAV plexus formation at 54 hpf. In the hypoxia-induced rupture phenotype, we counted the number of vascular disconnections in a 10 somite-long trunk area for each embryo at 54 hpf, examining the ISVs on both sides. To quantify the number of macrophages associated with vessels, we counted the number of macrophages close to blood vessels in a 10 somite-long trunk area for each embryo at 54 hpf, examining vessels on both sides of the embryo. Statistical analysis was performed using GraphPad software. Data presented in bar graphs represent mean±s.e.m. or s.d. *P* values were calculated by Student's *t*-test for single comparisons of normally distributed data (*P<0.05; **P<0.01; ***P<0.001; ****P<0.0001; NS, no significant changes observed).

### Data availability

The authors declare that all data supporting the findings of this study are available within the article and its Supplementary Information files or from the corresponding author upon reasonable request. Microarray data have been deposited in the GEO database under accession code GSE89117.

## Additional information

**How to cite this article:** Gerri, C. *et al*. Hif-1α regulates macrophage-endothelial interactions during blood vessel development in zebrafish. *Nat. Commun.* **8**, 15492 doi: 10.1038/ncomms15492 (2017).

**Publisher's note:** Springer Nature remains neutral with regard to jurisdictional claims in published maps and institutional affiliations.

## Supplementary Material

Supplementary Movie 1 DLAV plexus formation in a control embryo. Maximal intensity projections of time-lapse confocal images of a *Tg(kdrl:EGFP);Tg(mpeg:NTRmCherry)* control embryo starting at 30 hpf; lateral view. Arrowhead points to a macrophage colocalizing with EC sprouts.

Supplementary Movie 2 Macrophage ablation leads to the absence of DLAV plexus formation. Maximal intensity projections of time-lapse confocal images of a macrophage-ablated *Tg(kdrl:EGFP);Tg(mpeg:NTR-mCherry)* embryo starting at 30 hpf; lateral view.

Supplementary Movie 3 Macrophages appear to be involved in DLAV plexus formation in a WT embryo. Maximal intensity projections of time-lapse confocal images of a *Tg(kdrl:EGFP);Tg(mpeg:mCherry)* WT embryo starting at 30 hpf; dorsal view.

Supplementary Movie 4 Macrophages associate with unstable vessels just before their regression. Maximal intensity projections of time-lapse confocal images of a *Tg(kdrl:EGFP);Tg(mpeg:mCherry)* WT embryo starting at 58 hpf; lateral view.

Supplementary Movie 5 DLAV plexus formation fails to occur in *hif-1α* mutants. Maximal intensity projections of time-lapse confocal images of a *Tg(kdrl:EGFP);Tg(mpeg:mCherry)* *hif-1α* mutant starting at 30 hpf; dorsal view.

Supplementary Movie 6 DLAV plexus formation fails to occur in *hif-1α* mutants. Maximal intensity projections of time-lapse confocal images of a *Tg(kdrl:EGFP);Tg(mpeg:mCherry)* hif-1a mutant starting at 30 hpf; lateral view.

Supplementary Movie 7 Macrophages in vessel repair in a WT embryo. Maximal intensity projections of time-lapse confocal images of a 54 hpf *Tg(kdrl:EGFP);Tg(mpeg:mCherry)* WT embryo treated with DMOG starting at 48 hpf; lateral view. Yellow arrowhead points to a vessel rupture. White arrowhead points to recruited macrophages.

Supplementary Movie 8 In *hif-1α* mutants, macrophages do not appear to assist in vessel repair. Maximal intensity projections of time-lapse confocal images of a 54 hpf *Tg(kdrl:EGFP);Tg(mpeg:mCherry)* hif-1a mutant treated with DMOG starting at 48 hpf; lateral view.

Supplementary Movie 9 Macrophages closely associated with unstable vessels are *tnfa*:EGFPF+. 4 Maximal intensity projections of time-lapse confocal images of a 54 hpf *Tg(mpeg:mCherry);Tg(tnfa:EGFPF);Tg(kdrl:mCherry)* WT embryo treated with DMOG starting at 48 hpf; lateral view.

Supplementary Information Supplementary Figures, Supplementary Table.
