# Supplementary material for: Hif-1α regulates macrophage-endothelial interactions during blood vessel development in zebrafish
Source: Nat Commun. 2017 May 19;8:15492. doi: 10.1038/ncomms15492 (PMC5493593; doi:10.1038/ncomms15492)
Supplement: Supplementary Information — Supplementary Figures, Supplementary Table. [file ncomms15492-s1.pdf]

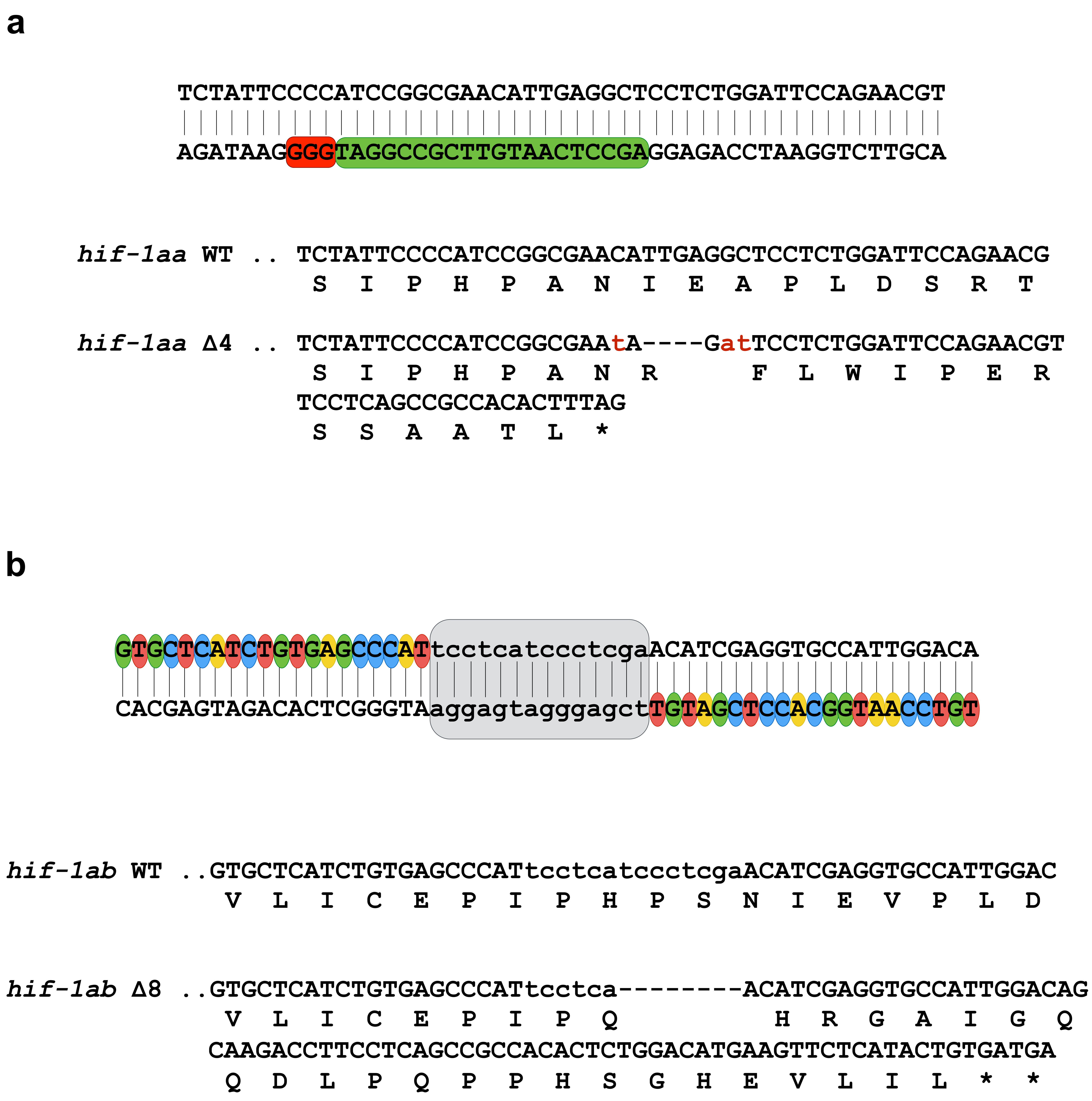

Supplementary Figure 1. Generation of *hif-1aa* and *hif-1ab* mutants.
(**a**) A CRISPR guide was designed to target the second PAS domain (exon 6) of *hif-1aa* (upper panel). The protospacer/target sequence is highlighted in green and the PAM sequence in red. The bottom panel shows the sequence alignment of the WT and *hif-1aa*^{bns89} alleles. The *hif-1aa*^{bns89} allele contains an indel mutation (−4) and 3 SNVs (single nucleotide variants) (red) leading to a premature stop codon after a 13 amino acid-long missense segment. (**b**) A TALEN guide was designed to target the first PAS domain (exon 6) of *hif-1ab* (upper panel). The spacer sequence is highlighted in grey surrounded by the left and right TAL repeats. The bottom panel shows the sequence alignment of the WT and *hif-1ab*^{bns90} alleles. The *hif-1ab*^{bns90} allele contains an indel mutation (−8) leading to a premature stop codon after a 23 amino acid-long missense segment.

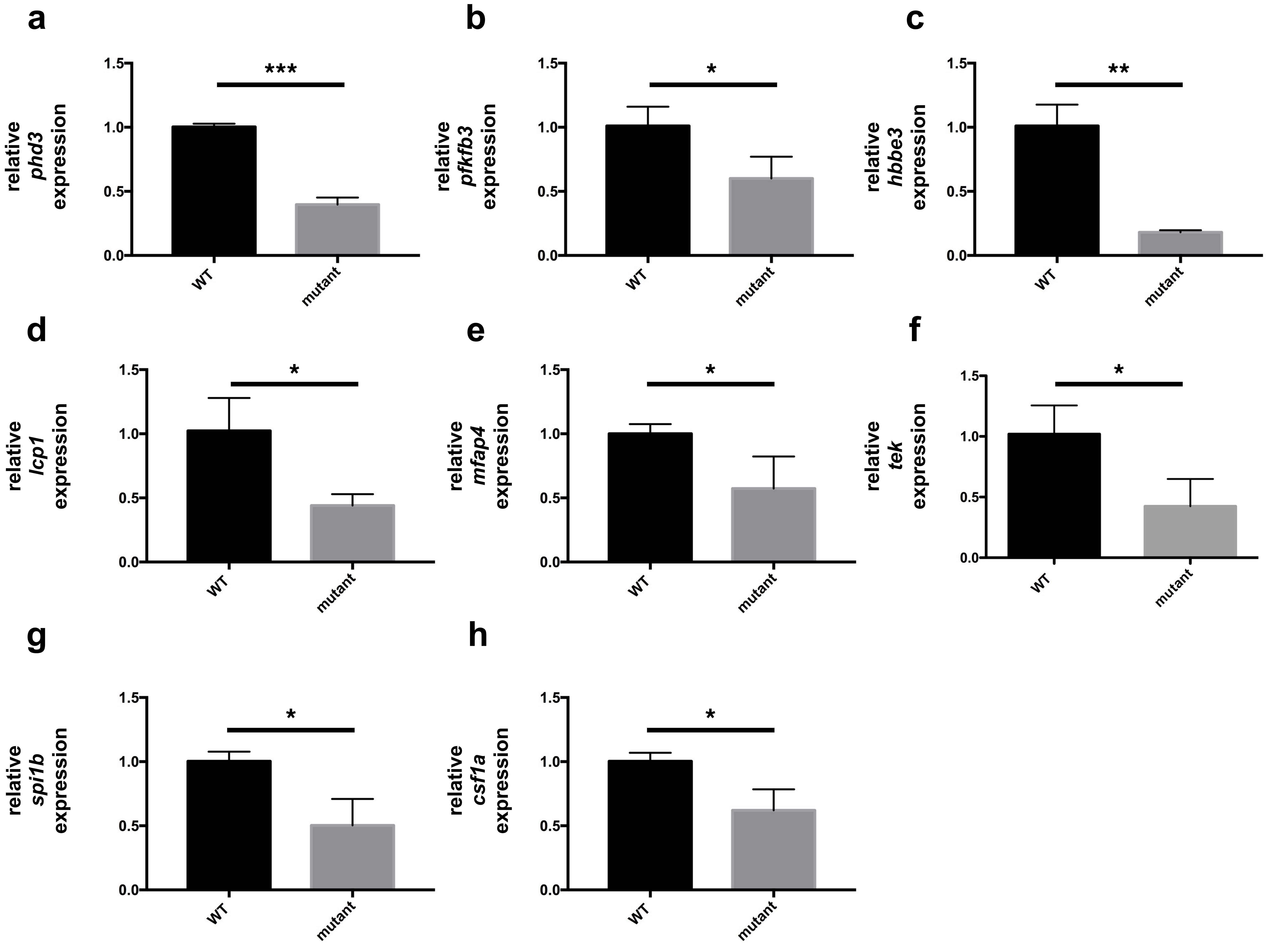

Supplementary Figure 2. qPCR analyses.

(**a-h**) qPCR analyses in 50 hpf WT siblings and *hif-1α* mutants after hypoxia chamber treatment. Values represent mean ± s.d., *n* = 3 biological replicates, (*$P < 0.05$; **$P < 0.01$; ***$P < 0.001$; *t*-test). Ct values for each gene in WT samples: *18S* = 16,47; *phd3* = 22,24; *pfkfb3* = 23,48; *hbbe3* = 17,19; *lcp1* = 23,30; *mfap4* = 21,24; *tek* = 28,92; *spi1b* = 25,00; *csf1a* = 23,14.

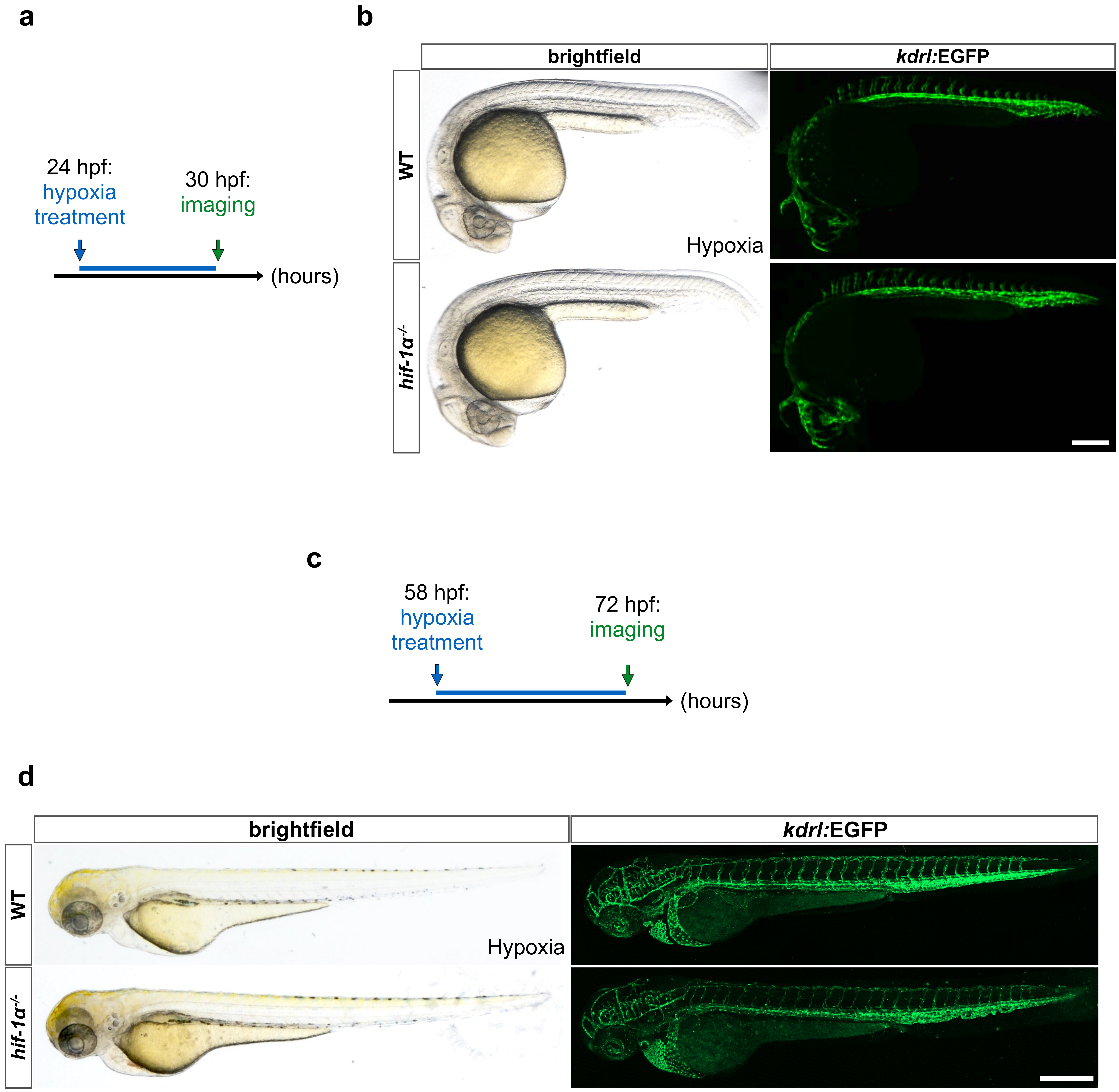

Supplementary Figure 3. *hif-1α* mutants do not show vascular anomalies at 30 hpf, and the phenotypes observed at 54 hpf do not appear more severe at 72 hpf.
(**a**) Schematic representation of the experiment shown in b. (**b**) Representative brightfield images and maximal intensity projections of confocal z-stacks of 30 hpf *Tg(kdrl:EGFP)* WT sibling and *hif-1α*$^{-/-}$ embryos after hypoxia chamber treatment for 6 hours starting at 24 hpf; lateral views. n = 3 different clutches. (**c**) Schematic representation of the experiment shown in d. (**d**) Representative brightfield images and maximal intensity projections of confocal z-stacks of 72 hpf *Tg(kdrl:EGFP)* WT sibling and *hif-1α*$^{-/-}$ larvae after hypoxia chamber treatment for 14 hours starting at 58 hpf; lateral views. n = 3 different clutches. Scale bars, 200 μm.

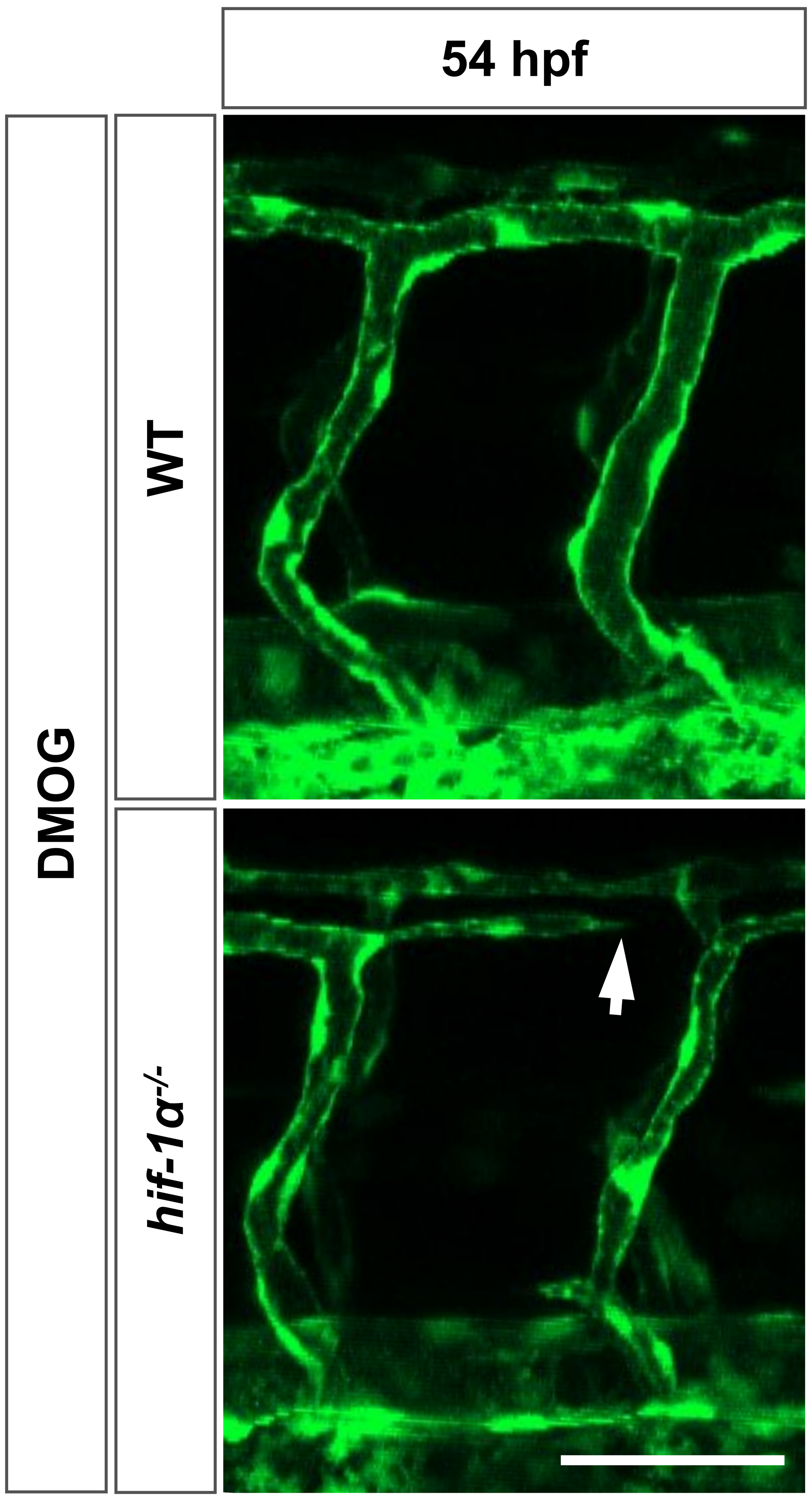

Supplementary Figure 4. *hif-1a* mutants exhibit vascular disconnections. Maximal intensity projections of confocal z-stacks of 54 hpf *Tg(kdrl:EGFP)* WT sibling and *hif-1α*$^{-/-}$ embryos after DMOG treatment for 6 hours starting at 48 hpf. Arrow points to a blood vessel rupture. *n* = 3 different clutches. Scale bar, 50 µm.

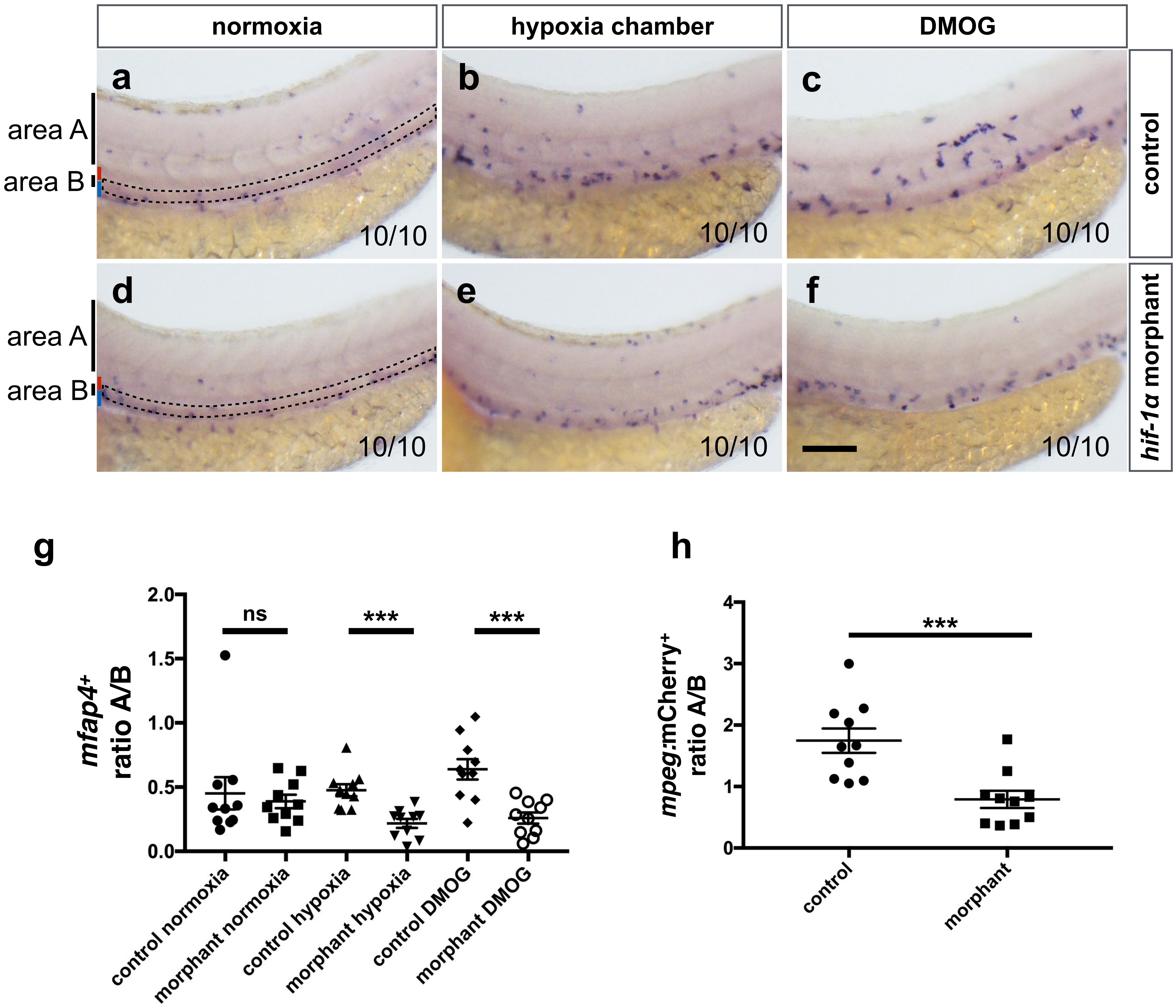

Supplementary Figure 5. ***hif-1α*** **morphants phenocopy the macrophage mobilization defects observed in** ***hif-1α*** **mutants.**

(**a-f**) Brightfield images of WISH for *mfap4* expression in 54 hpf control morphants and *hif-1α* morphants in normoxia, after hypoxia chamber or DMOG treatment for 6 hours starting at 48 hpf; lateral views. Area A is situated outside the AGM and area B represents the AGM region (outlined). Red bar marks approximate size of the dorsal aorta and blue bar that of the posterior cardinal vein. n = 10 embryos from 3 different clutches. Scale bar, 100 μm. (**g**) Quantification of macrophage mobilization from the AGM based on *mfap4* WISH experiments, showing the ratio of macrophage number in area A to macrophage number in area B. (**h**) Quantification of macrophage mobilization from the AGM of 54 hpf *Tg(kdrl:EGFP);Tg(mpeg:mCherry)* control morphants and *hif-1α* morphants in normoxia. Bars represent mean ± s.e.m., n = 10 embryos from 3 different clutches, (***$P < 0.001$; ns: no significant changes observed; *t*-test).

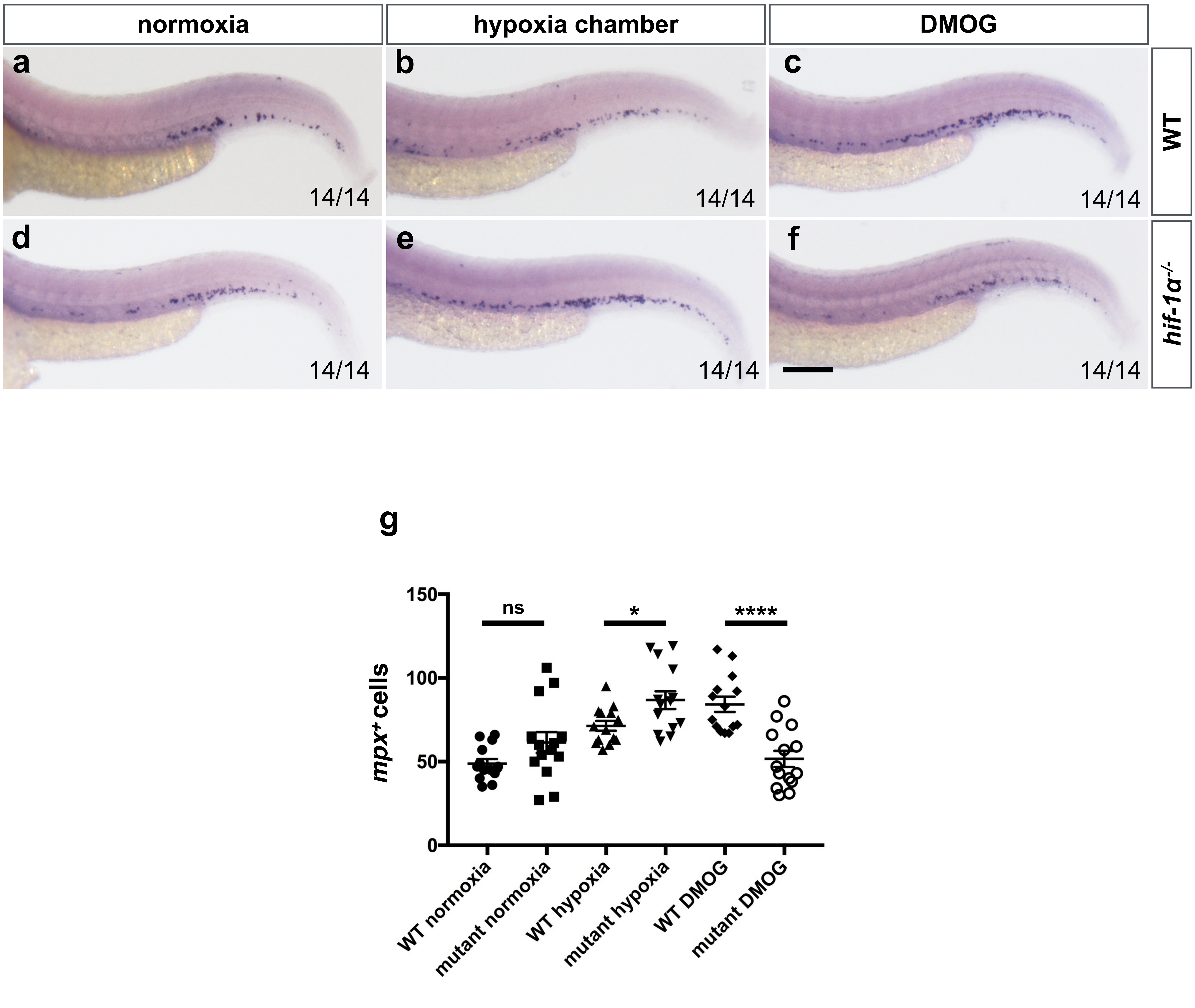

Supplementary Figure 6. Neutrophil quantification in *hif-1a* mutants.
(**a-f**) Brightfield images of WISH for *mpx* expression in 54 hpf WT siblings and *hif-1a* mutants in normoxia, after hypoxia chamber or DMOG treatment for 6 hours starting at 48 hpf; lateral views (**g**) Quantification of neutrophil number at 54 hpf. Bars represent mean ± s.e.m., $n = 14$ embryos from 3 different clutches, (*$P < 0.05$; ****$P < 0.0001$; ns: no significant changes observed; *t*-test). Scale bar, 100 µm.

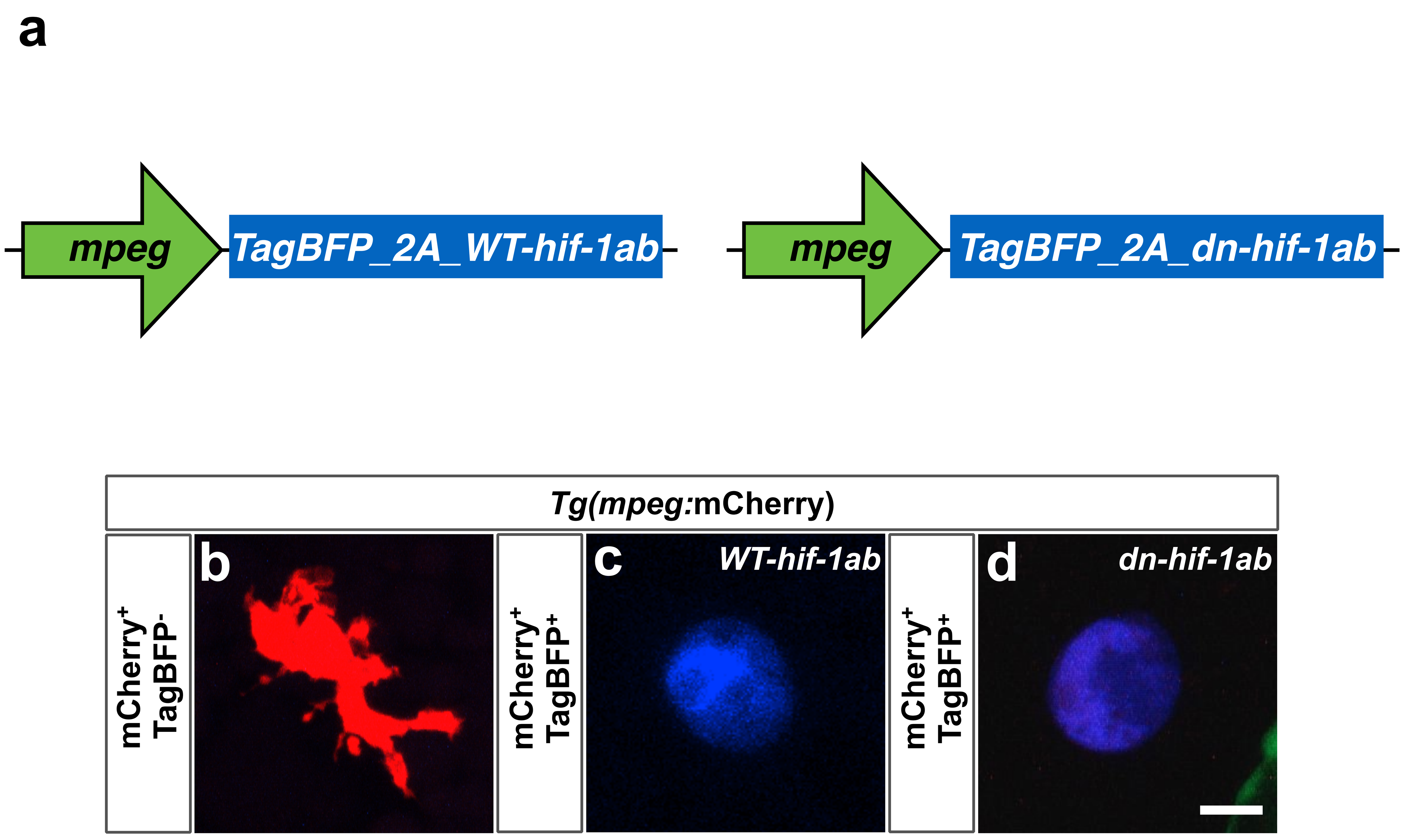

Supplementary Figure 7. Overexpression of *WT-Hif-1ab* and *dn-Hif-1ab* affects macrophage morphology.

(**a**) Plasmids used to mosaically express *WT-hif-1ab* and *dn-hif-1ab*. (**b**) Maximal intensity projections of confocal z-stacks of *Tg(mpeg:mCherry)* WT macrophage at 54 hpf. (**c,d**) Maximal intensity projections of confocal z-stacks of a *mpeg:*TagBFP_2A_WT-hif-1ab $^{+}$;*Tg(mpeg:*mCherry)$^{+}$ *hif-1α* mutant macrophage and of a *mpeg:*TagBFP_2A_dn-hif-1ab $^{+}$;*Tg(mpeg:*mCherry)$^{+}$ WT macrophage at 54 hpf. Scale bar, 5 μm.

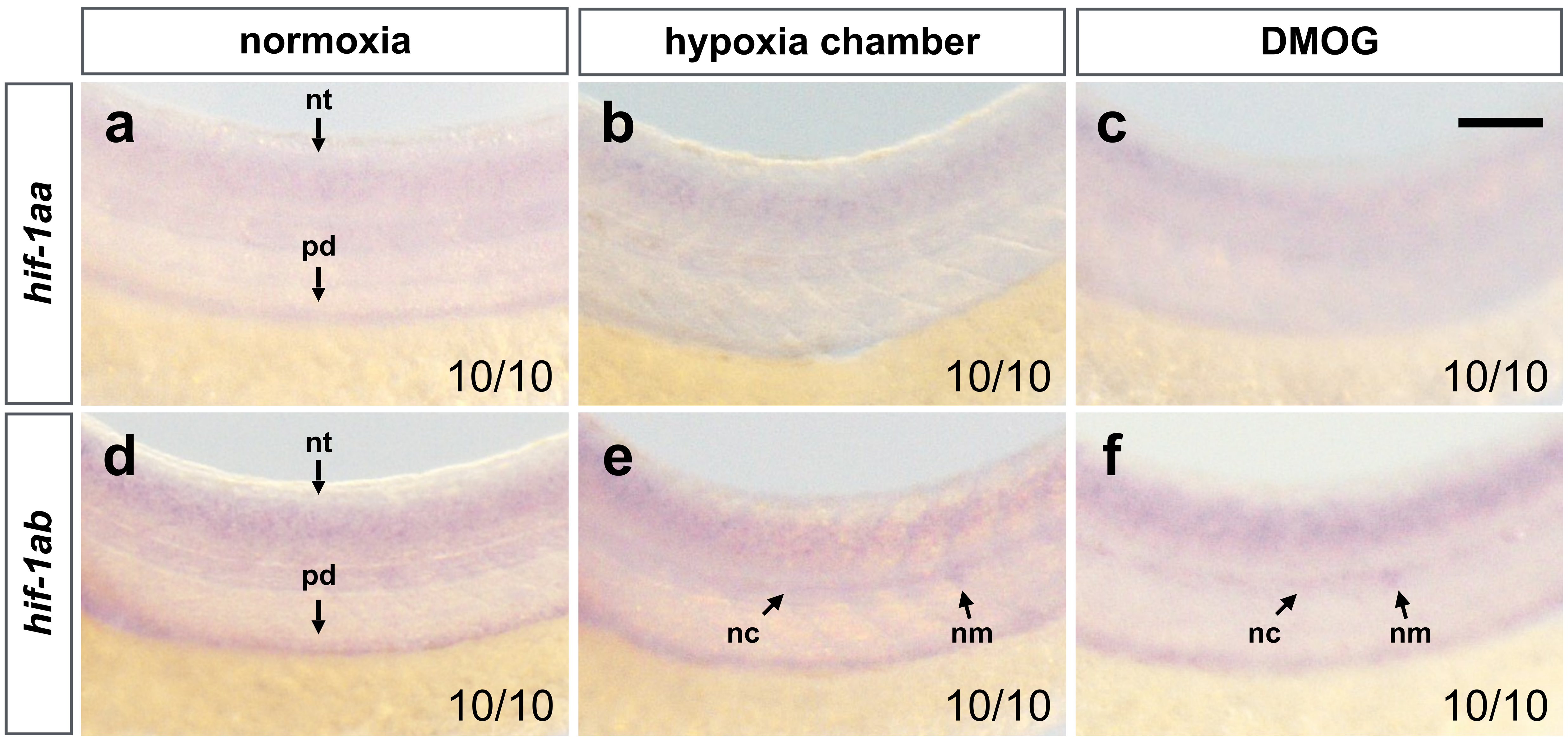

Supplementary Figure 8. ***hif-1aa*** **and** ***hif-1ab*** **are not highly expressed in ECs at 54 hpf.** (**a-c**) Brightfield images of WISH for *hif-1aa* expression in 54 hpf WT embryos in normoxia, after hypoxia chamber or DMOG treatment for 6 hours starting at 48 hpf; lateral views. (**d-f**) Brightfield images of WISH for *hif-1ab* in 54 hpf WT embryos in normoxia, after hypoxia chamber or DMOG treatment for 6 hours starting at 48 hpf; lateral views. nt: neural tube; pd: pronephric duct; nc: notochord; nm: neuromast. n = 10 embryos from 3 different clutches. Scale bar, 50 μm.

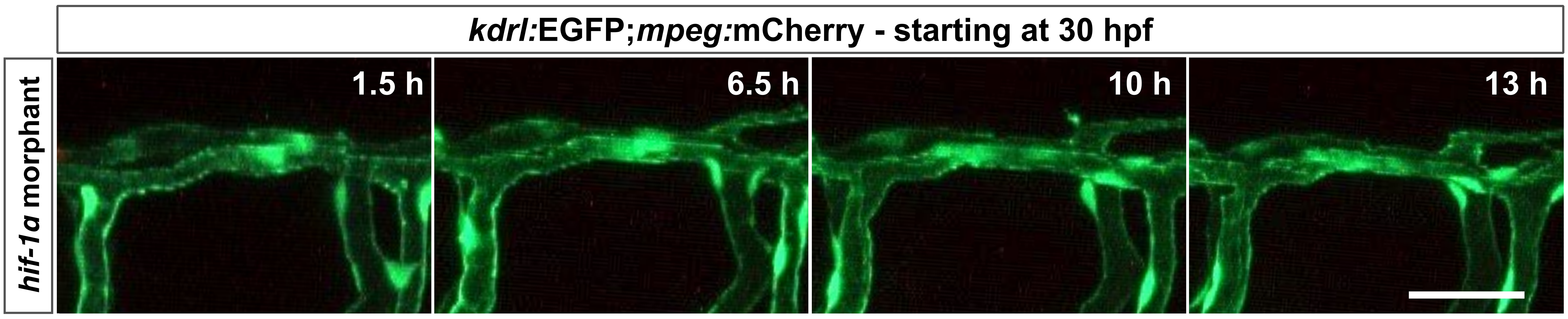

Supplementary Figure 9. DLAV plexus formation is absent in *hif-1α* morphants.
Maximal intensity projections of time-lapse confocal images of a *Tg(kdrl:EGFP);Tg(mpeg:mCherry)* *hif-1α* morphant starting at 30 hpf; lateral views. $n = 3$ different clutches. Scale bar, 50 μm.

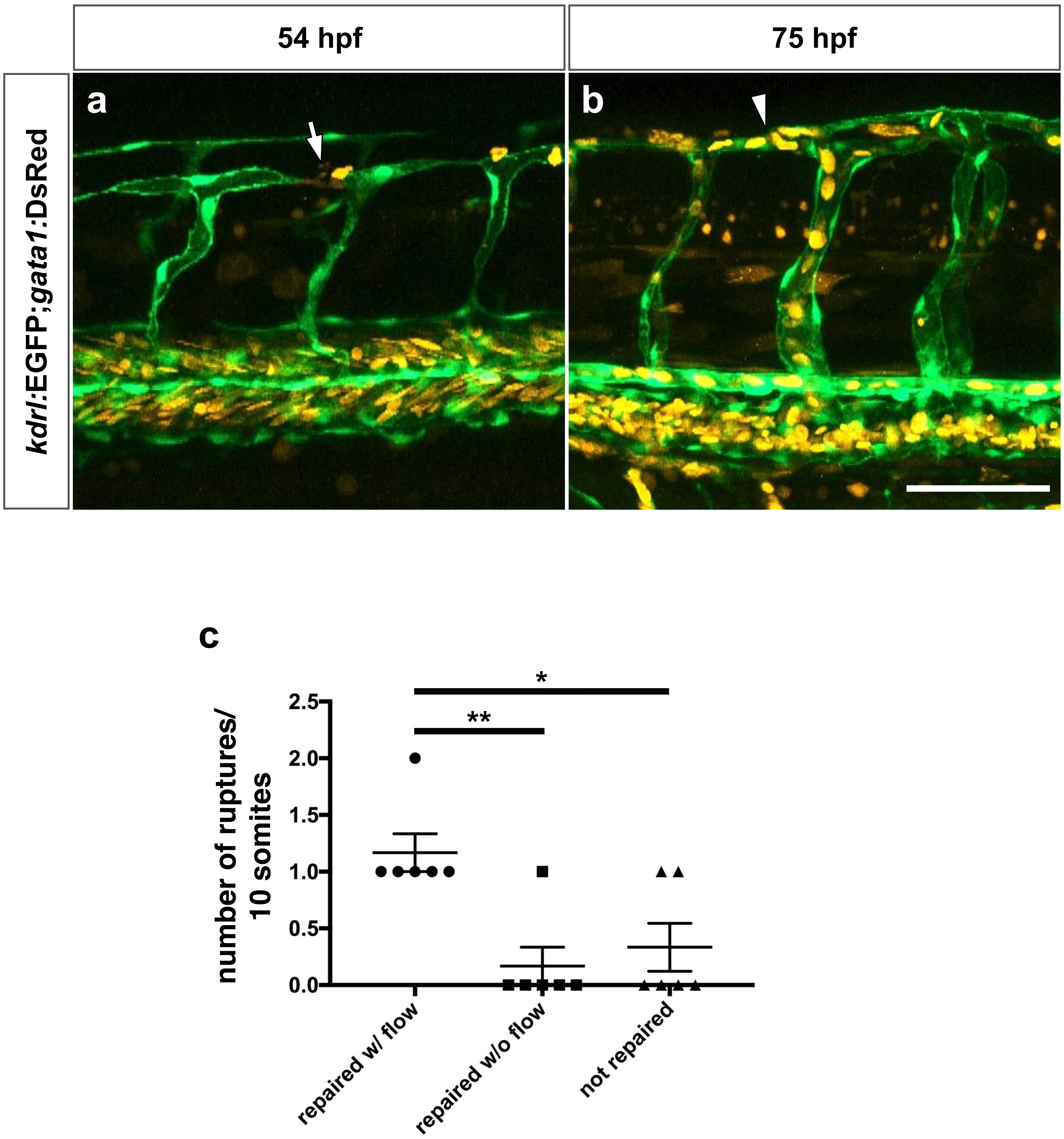

Supplementary Figure 10. Repaired blood vessels in WT embryos regain blood flow. (**a**) Maximal intensity projections of confocal z-stacks of a blood vessel rupture in a 54 hpf *Tg(kdrl:EGFP);Tg(gata1:DsRed)* WT embryo after DMOG treatment for 6 hours starting at 48 hpf. (**b**) Maximal intensity projections of confocal z-stacks of the same blood vessel in the same embryo after repair. Lateral views; white arrow points to a ruptured vessel showing erythrocyte leakage, white arrowhead points to repaired vessel having regained flow. (**c**) Quantification of ruptured vessels subsequently repaired and showing presence (w/) or absence (w/o) of flow as well as those not repaired; data collected at 75 hpf. Bars represent mean ± s.e.m., $n = 6$ from 3 different clutches. (*$P < 0.05$; **$P < 0.01$; *t*-test). Scale bar, 50 μm.

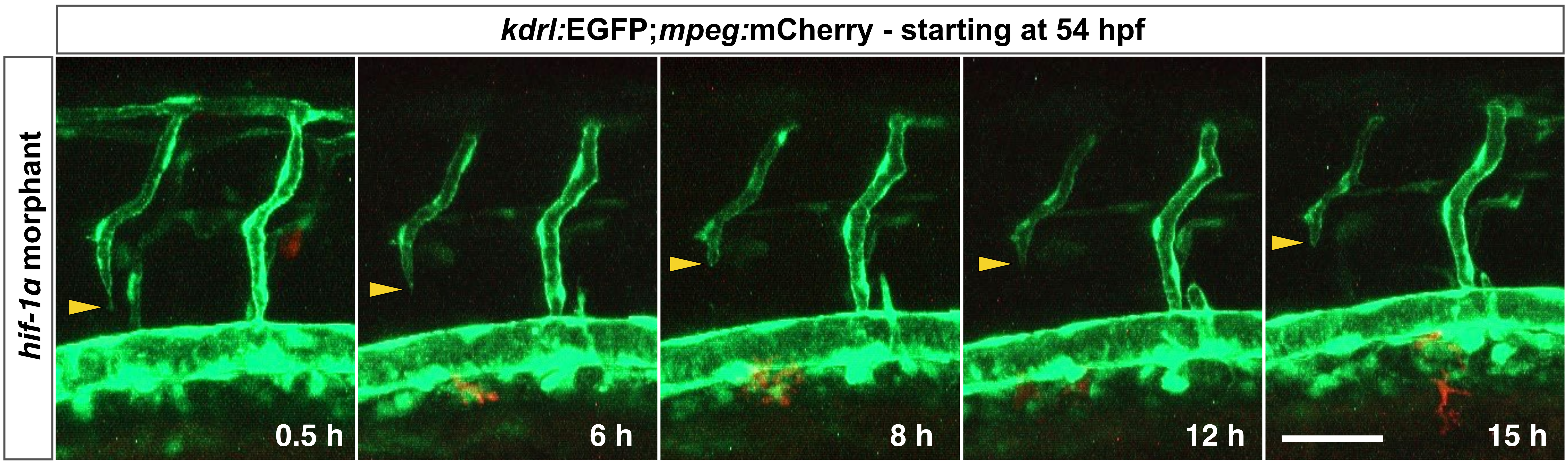

Supplementary Figure 11. Macrophages do not appear to assist in vessel repair in *hif-1a* morphants.

Maximal intensity projections of time-lapse confocal images of a *Tg(kdrl:EGFP);Tg(mpeg:mCherry) hif-1a* morphant treated with DMOG starting at 48 hpf; lateral views. Yellow arrowheads point to ruptured vessel. *n* = 3 different clutches. Scale bar, 50 μm.

hif-1aa CRISPR target sequence	5' AGCCTCAATGTTCGCCGGAT 3'

Supplementary Table 1. CRISPR target sequence used to generate *hif-1aa* mutants.
